# A universal power law for modelling the growth and form of teeth, claws, horns, thorns, beaks, and shells

**DOI:** 10.1186/s12915-021-00990-w

**Published:** 2021-03-30

**Authors:** Alistair R. Evans, Tahlia I. Pollock, Silke G. C. Cleuren, William M. G. Parker, Hazel L. Richards, Kathleen L. S. Garland, Erich M. G. Fitzgerald, Tim E. Wilson, David P. Hocking, Justin W. Adams

**Affiliations:** 1 School of Biological Sciences, Monash University, Melbourne, Victoria 3800 Australia; 2 Geosciences, Museums Victoria, Melbourne, Victoria 3001 Australia; 3 School of Mathematical Sciences, Monash University, Melbourne, Victoria 3800 Australia; 4 Biomedicine Discovery Institute, Monash University, Melbourne, Victoria 3800 Australia

**Keywords:** Shape generation, Morphogenesis, Differential growth, Vertebrates, Teeth, Logarithmic spiral, Evo-devo, Power law, Power cascade, Power cone

## Abstract

**Background:**

A major goal of evolutionary developmental biology is to discover general models and mechanisms that create the phenotypes of organisms. However, universal models of such fundamental growth and form are rare, presumably due to the limited number of physical laws and biological processes that influence growth. One such model is the logarithmic spiral, which has been purported to explain the growth of biological structures such as teeth, claws, horns, and beaks. However, the logarithmic spiral only describes the path of the structure through space, and cannot generate these shapes.

**Results:**

Here we show a new universal model based on a power law between the radius of the structure and its length, which generates a shape called a ‘power cone’. We describe the underlying ‘power cascade’ model that explains the extreme diversity of tooth shapes in vertebrates, including humans, mammoths, sabre-toothed cats, tyrannosaurs and giant megalodon sharks. This model can be used to predict the age of mammals with ever-growing teeth, including elephants and rodents. We view this as the third general model of tooth development, along with the patterning cascade model for cusp number and spacing, and the inhibitory cascade model that predicts relative tooth size. Beyond the dentition, this new model also describes the growth of claws, horns, antlers and beaks of vertebrates, as well as the fangs and shells of invertebrates, and thorns and prickles of plants.

**Conclusions:**

The power cone is generated when the radial power growth rate is unequal to the length power growth rate. The power cascade model operates independently of the logarithmic spiral and is present throughout diverse biological systems. The power cascade provides a mechanistic basis for the generation of these pointed structures across the tree of life.

**Supplementary Information:**

The online version contains supplementary material available at 10.1186/s12915-021-00990-w.

## Background

The discovery of general models and mechanisms that create the phenotypes of organisms is a major goal of evolutionary developmental biology [1–5]. Very few such fundamental growth patterns exist, including logarithmic spiral growth [6, 7]. These growth patterns are important because they significantly influence the diversity of life by making some phenotypes very common while constraining or even prohibiting others, essentially favouring specific evolutionary trajectories [1, 8–13].

**Fig. 1:**
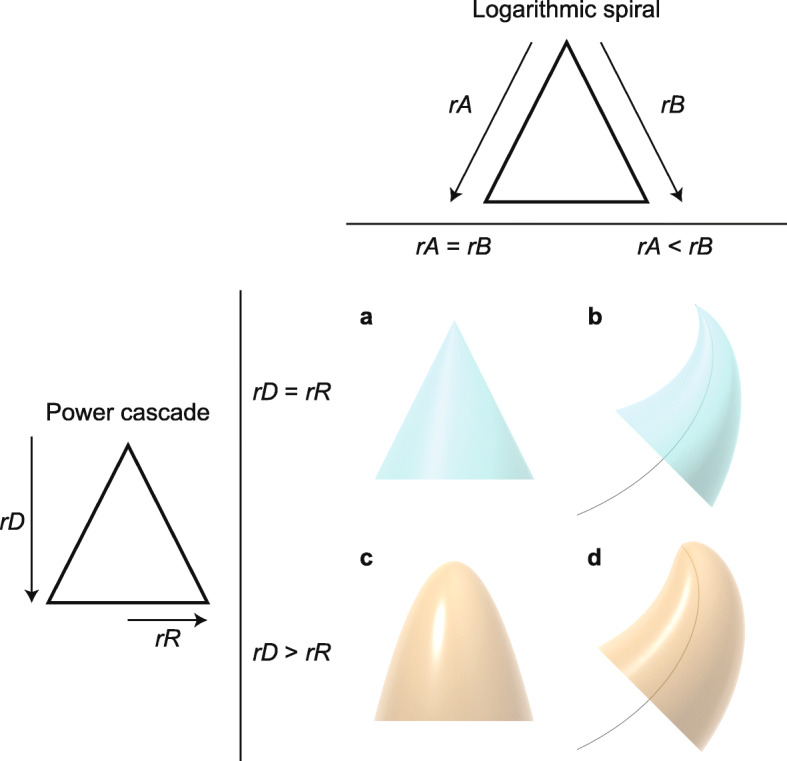
Generative models for shapes of teeth and other pointed structures, showing the effects of relative growth rates on shape. Logarithmic spiral (top): if the rates of growth of the two sides of the structure (*rA* and *rB*) are equal, a symmetrical structure such as a cone is produced (**a**). If the rate of growth on one side is lower (e.g. *rA* < *rB*), then the structure curves to follow a logarithmic spiral (black curved line) (**b**). Power cascade (left): when the power growth rate of the distance from the tip (*rD*) is equal to the growth rate of the radius (*rR*), then a cone is produced (**a**). When *rR* is less than *rD*, a power cone is generated (**c**). Here, *rD* = 2*rR*, generating a paraboloid. Both of these inequalities in growth rates can be combined to form a power cone curving along a logarithmic spiral, or a power spiral (**d**)

The vertebrate dentition, with its panoply of morphological diversity, is a superb system in which to investigate models of growth. All vertebrate teeth grow from the tip downwards towards the base to form the main body and individual cusps of each tooth. Teeth are often described as being ‘conical’ [14, 15]—this term may refer to the strict mathematical shape of a cone (Fig. 1a) or perhaps a single-pointed structure that folds down on all sides. While we have made great strides in determining the genetic influences on cusp formation and variation [16, 17], currently, we do not know the main determinants of cusp shape.

Following the discovery of the logarithmic spiral [18], in 1659, Sir Christopher Wren [19] first proposed that shells grow as a cone expanding along a logarithmic spiral (Fig. 1a, b), so that the trajectory of the midline of the shell forms a logarithmic spiral. This approach has since been used to model shell growth [7, 20]. Thompson [6] concluded that teeth follow this conical pattern of growth; here, we will test the suggestion of Wren [19] and Thompson [6] that teeth grow in the shape of a cone. At the same time, we strive to ascertain whether there are high-level, simple models governing how teeth grow and to determine if these patterns extend beyond the dentition to other pointed structures in nature.

## Results

### Power cascade model simulates tooth growth

**Fig. 2:**
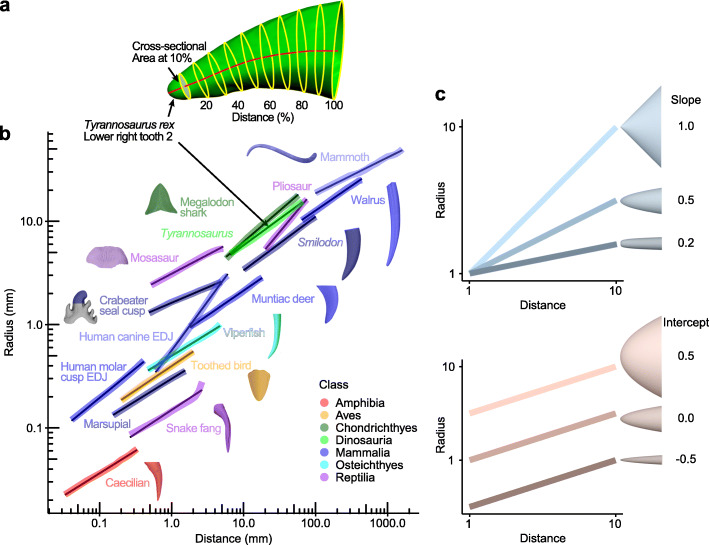
Vertebrate teeth grow following the power cascade model, showing linear change in log *Radius* with log *Distance* from tip. **a** Measurement of *Radius* and *Distance* for *Tyrannosaurus rex* (UWBM 99000) lower right tooth 2 (shown in light green in **b**), which fits a linear model with *R*^2^ = 0.997. *Radius* = √ (cross-sectional area/π). **b** Teeth from all vertebrate groups show a linear pattern on log-log axes. Linear regression gives *R*^2^ > 0.994 for all teeth shown, other than snake fang where *R*^2^ = 0.954 (*R*^2^ = 0.999 excluding base 20%). EDJ, enamel-dentine junction. **c** Power cones vary depending on *Slope* (from conical to blunt) and *Intercept* (from wide to narrow) of the *Log Radius-Distance* plot

We represent the shape of the tooth in the manner in which it grows, from tip to base, by measuring the rate of lateral expansion of the tooth as the length increases. To measure this rate for a tooth, we take a 3D digitised surface of the tooth and place 10 equally spaced cross-sections perpendicular to its midline (Fig. 2a). The average radius of each cross-section is *Radius* = √ (cross-sectional area/π). We then plot log_10_*Distance* from the tip against log_10_*Radius*. The example *Tyrannosaurus rex* tooth illustrated in Fig. 2a shows a very close relationship to a straight line (Fig. 2b), fitting a linear model with *R*^2^ = 0.997.

All other teeth measured show a strong linear or close to linear relationship between log(*Distance*) and log(*Radius*) (here termed *Log Distance-Radius* plots; Fig. 2b; *R*^2^ range 0.9654–0.9998; see Additional file 1: Tables S1-S5 for sample sizes). This appears to indicate a general model of growth for single-cusped teeth, including bony fish, sharks, amphibians, reptiles, non-avian dinosaurs, birds, and mammals (Additional file 1: Figure S1).

The linear relationship, explicitly written log_10_(*Radius*) = *Slope* × log_10_(*Distance*) + *Intercept*, allows the growth and shape of a tooth to be characterised by its *Slope* and *Intercept*. This relationship can be written as *Radius* = 10^*Intercept*^ × *Distance*^*Slope*^. Therefore, the relationship is a ‘power law’ [21] (monomial) with varying exponent (*Slope*) and multiplier (10^*Intercept*^).

When *Slope* = 1, there is a linear relationship between the raw measurements of *Distance* and *Radius*. Revolving this straight line around the *x*-axis to form a surface of revolution generates a cone (Fig. 1a; Additional file 1: Figure S2a). This shape is the cone expected by Wren [19]. When *Slope* = 0.5, the surface of revolution is a paraboloid (Fig. 1c; Additional file 1: Figure S2a). Values below 0.5 are increasingly blunt at the very tip. The shape of the unicuspid teeth measured above therefore matches the surface of revolution of a power function, which we call a ‘power cone’ and is the same as the ‘power series’ of airplane nose cone designs [22]. We will use the term ‘cone’ to refer to only the straight-sided conventional cone, while ‘power cone’ is the more general shape with a profile of varying curvature. Because *Slope* is always less than 1 (range 0.25–0.95), teeth are therefore not cone-shaped and do not match the conical model of Wren [19]. In order to describe the folding of the tooth shape cascading down from the tip of the tooth according to the power function, we term this model the ‘power cascade’. The power cascade defines a new family of shapes that vary in *Slope* and *Intercept*.

The *Intercept* represents a scaling factor for the width of the tooth, with higher values resulting in wider teeth for the same length (Additional file 1: Figure S2b). The right-hand end of the *Log Distance-Radius* curve represents the maximum length of the tooth (Additional file 1: Figure S2c). Since all teeth plotted in Fig. 2 have been measured at ten equally spaced points along the tooth, the first distance for each tooth (x minimum) is 1/10 of the maximum distance (x maximum), and so these lines are the same length along the log-scaled *x*-axis (the only exception being the mammoth tusk; Additional file 1: Figure S2c). Ten intervals appears to be minimally required to represent the shape of a tooth; however, more points, whether or not equally spaced, can be used (see Additional file 1: Figure S3 for alternative sampling intervals) and do not substantially affect the calculation of *Slope* or *Intercept* (see Supplementary Discussion).

### Tooth crowns, rows, and cusps follow the power cascade

**Fig. 3:**
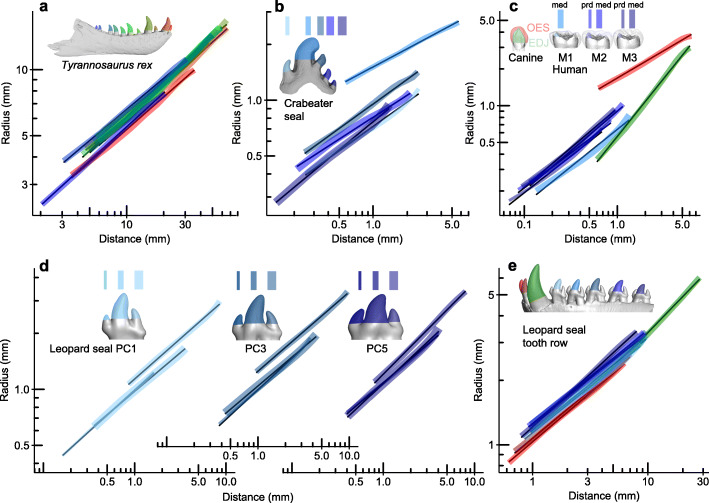
Teeth, tooth rows and cusps follow the power cascade. **a** Eleven teeth in the jaw of *Tyrannosaurus rex* (UWBM 99000): 0.992 > *R*^2^ > 0.999. **b** Separate cusps on a crabeater seal *Lobodon carcinophaga* (NMV C7385) postcanine 3: 0.990 > *R*^2^ > 0.998. **c** Human *Homo sapiens* lower enamel-dentine junction (EDJ) and outer enamel surface (OES) canine and separate EDJ cusps on three lower molars (protoconid (prd) of M2 and M3, thin line; metaconid (med) of M1, M2 and M3, thick line): 0.984 < *R*^2^ < 0.998. **d** All three cusps of the postcanines (PC) 1, 3 and 5 of the leopard seal *Hydrurga leptonyx* (NMV C31561): 0.995 < *R*^2^ < 1.000. **e** Two incisors, one canine and central cusps of all five postcanines of the leopard seal: 0.997 < *R*^2^ < 1.000

The junction between the enamel and dentine layers in a tooth initially forms as the interface between the epithelium and mesenchyme during development [23]. In most vertebrates, the enamel or enameloid has an equal thickness over the tooth surface, and so the outer enamel surface is an adequate approximation of the initial shape of the developing tooth. In some groups such as hominids, the enamel is of uneven thickness over the tooth (modelled by [24]). However, both the enamel-dentine junction (EDJ) and the outer enamel surface of the human canine show a tight fit to the power cascade (*R*^2^ > 0.997), although with different slopes (Fig. 3c).

Many reptiles and marine mammals have rows of unicuspid teeth along each jaw, sometimes showing variation in tooth shape along the row [25]. To investigate whether the power cascade sufficiently represents growth in all teeth within a row, we measured 11 teeth in the lower jaw of the theropod dinosaur *Tyrannosaurus rex*. All teeth are represented by the power function (0.992 < *R*^2^ < 0.999; Fig. 3a), with *Slope* between 0.47 and 0.54.

Most mammal teeth have multiple cusps on the surface. Each of these cusps is generated from the position of an enamel knot that forms a local maximum of the epithelium-mesenchyme interface during early tooth development [16, 23]. Leopard seal *Hydrurga leptonyx* postcanine teeth are trident-shaped, with a large central cusp and smaller anterior and posterior cusps (Fig. 3d). Measuring each of these cusps separately shows a linear *Log Distance-Radius* relationship for each cusp. The *Slope* and *Intercept* are similar for all five postcanine teeth, with the central cusp tending to have a higher *Intercept* but similar *Slope* (Fig. 3d). More rounded cusps also show power cascade tooth growth, as seen in the wave-like shapes of the crab-eater seal *Lobodon carcinophaga* postcanine teeth (Fig. 3b) and in the enamel-dentine junction of the individual cusps of human molars (Fig. 3c). The entire heterodont tooth row of the leopard seal shows strong linear patterns in the incisors, canines, and main postcanine cusps (*R*^2^ > 0.997; Fig. 3e).

### Power cascade model can predict tooth length and age

The attributes of the power cascade model have important implications for the growth and characterisation of teeth. Teeth are often worn or broken at the tip through use in life or during preservation and fossilisation. If teeth generally show growth according to a power cascade model, we can use this to estimate how much of the tip of a tooth has been lost. By sequentially adding length to the tip and fitting a linear model, the distance corresponding to the original tooth length should be closest to linear and have the highest *R*^2^ value for the regression. To demonstrate this concept, we can artificially remove part of the tip of a leopard seal canine and then estimate how much has been lost (Additional file 1: Figure S4b,c), resulting in an overestimate of 5.8 to 8.0% of the original length of the tooth (or less than 5% overestimate if accounting for apparent minor changes in growth early in tooth formation; see Supplementary Information). This method has been used to reconstruct the missing tip of some teeth in Fig. 2 such as the pliosaur fossil tooth (Additional file 1: Figure S4d).

A new method for estimating the age of mammals with ever-growing teeth, such as elephants and rodents, can be derived from this ability to reconstruct tooth length. Through use, a substantial amount of an ever-growing tooth is worn away (e.g. tusk wear on trees and gnawing in rodents), and so the complete length of the tooth must first be estimated using the power cascade. Then, we can use the rate of tooth growth (microns per day, based on crown extension rate [26, 27]) to calculate the length of time taken to grow the tooth. The result is a minimum estimate of the age of the individual.

**Fig. 4:**
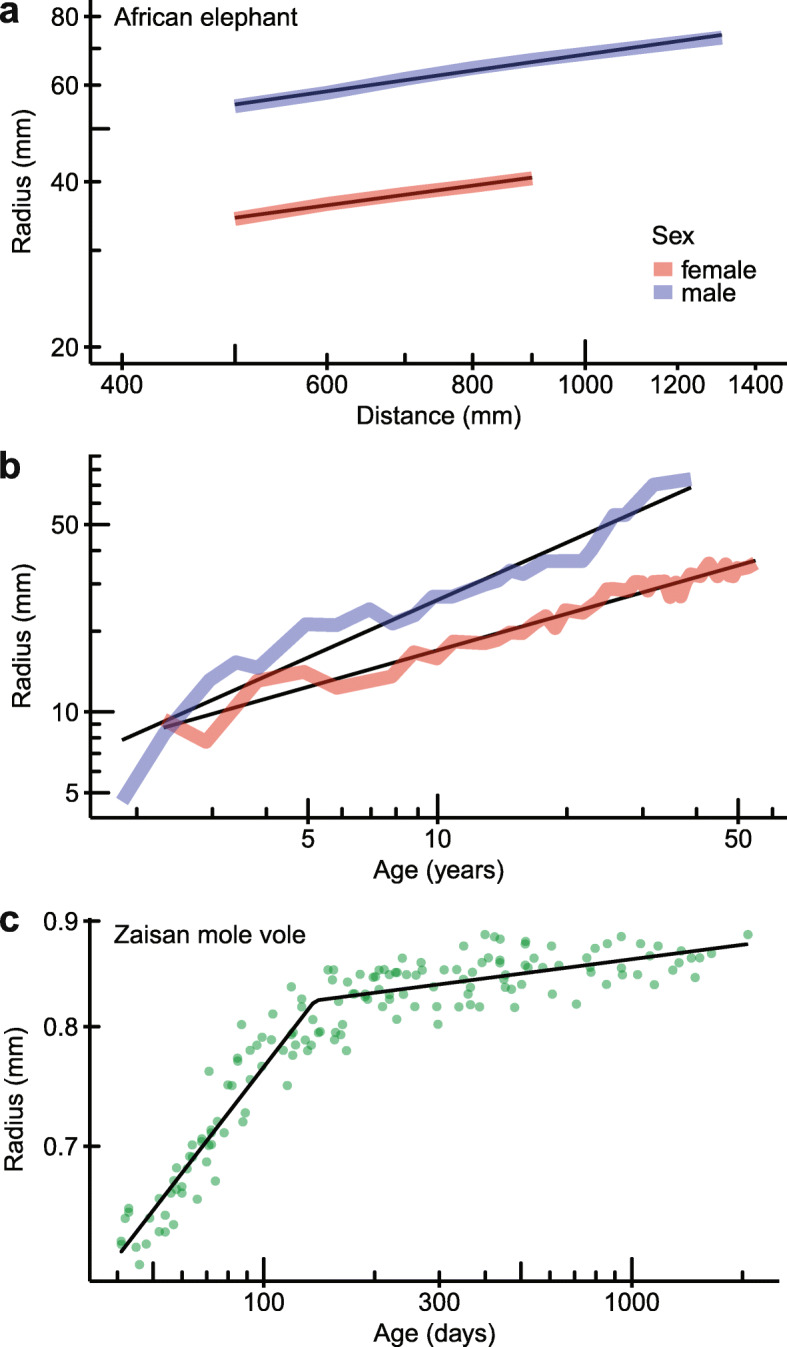
The power cascade can predict the age of mammals with ever-growing teeth. **a** Tusk *Radius* increases with *Distance* for male (USNM 49759 right tusk) and female (USNM 165501 right tusk) African elephants *Loxodonta africana* [29] following the power cascade. *Intercept* differs between male (1.229; *R*^2^ = 0.991) and female (1.050; *R*^2^ = 0.995) tusks and therefore could be used to determine sex of isolated tusks. *Slope* is similar for both sexes (0.303 and 0.287, respectively). **b** Tusk *Radius* at the lip line increases with *Age* according to the power cascade. *SlopeAge* differs between males (0.712; *R*^2^ = 0.927) and females (0.453; *R*^2^ = 0.957). Mean tusk radius in yearly age classes for 247 females and 99 males [28]. **c** Zaisan mole vole *Ellobius tancrei* incisor *Radius* increases with *Age* according to the power cascade during juvenile growth, and then dramatically decreases growth rate in adulthood. Segmented linear regression of log variables shown as black line. Juvenile *SlopeAge* = 0.231, *R*^2^ = 0.875. Incisor radius for 158 mole voles with known age [30]

In African elephants *Loxodonta africana* [28], *Radius* increases with *Distance* along the tusk following the power cascade relationship. *Intercept* is higher in males than females (Fig. 4a), thereby giving the ability to determine sex from an isolated tusk according to the rate of increase in radius. As expected, *Radius* at the base of a tusk increases with *Age* in years according to the power cascade (Fig. 4b), therefore giving an estimated *Age* for a given tusk *Radius*. A similar power cascade pattern is found in the incisors of the Zaisan mole vole *Ellobius tancrei* [30] during juvenile growth (Fig. 4c) but at a much smaller scale. While incisor circumference or width have been used to estimate age previously based solely on regression [30, 31], the power cascade describes the underlying pattern, and allows for estimation of age from growth parameters alone. Additional work on the limitations and accuracy of predictions derived from this approach is currently underway.

Close inspection of the *Log Distance-Radius* curves in Fig. 2 shows some deviations from the expected linear pattern of the power cascade. This is most obvious in the acrodont snake teeth, where the tooth fuses to the bone rather than sitting in a bony socket. The initial shaft of the tooth follows the power cascade model, but close to its base, the radius increases faster than expected, deviating from linear. This is likely due to additional widening of the dental epithelium at the base of the tooth to aid the fusion of the tooth to the bone in these snakes. Deviation from the power cascade pattern may also occur as the developing tooth is curved to fit within the jaw before mineralisation has occurred.

Another apparent cause of deviation from the power cascade is the presence of grooves running down part of the length of the tooth, such as those found in snake fangs (Fig. 2) and felid canines (Additional file 1: Figure S1). The infolding of the grooves reduces the cross-sectional area and so appears to cause deviation from the expected linear pattern. Wear on the tip or lateral surface of the teeth will also cause deviation, and so slightly worn or broken teeth were only included where they could be confidently reconstructed.

### Tooth shape is defined by power cascade morphospace of *Slope* and *Aspect Ratio*

Power cones can be considered self-similar curves in that all curves of the same power can be stretched or rescaled to be the same shape (using affine transformations of translation and scaling akin to self-affine fractals; Additional file 1: Figure S5). Therefore, the only distinguishing feature of teeth with the same *Slope* is the relative stretching of the curve, i.e. the *Aspect Ratio*, calculated as the maximum length divided by the maximum diameter (Additional file 1: Figure S2).

**Fig. 5:**
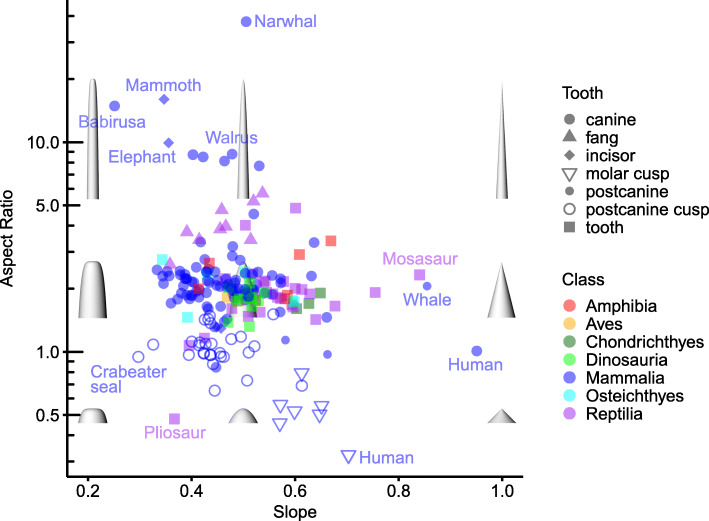
Morphospace of vertebrate teeth based on power cascade *Slope* and *Aspect Ratio*. *Slope* = 1 indicates conical shape, *Slope* = 0.5 is a paraboloid, while lower *Slope* are progressively more blunt power cones. *Aspect Ratio* is length of tooth divided by width at base. Colour indicates vertebrate Class; shape indicates tooth or cusp type. Models are shown for *Slope* values 0.2, 0.5, and 1.0 and *Aspect Ratio* values 0.5, 2, and 10

We can use these two key parameters of the power cascade to define dimensions of a morphospace that illustrates the range of combinations found in vertebrate teeth (Fig. 5). Squat shapes are at the bottom of the graph—these are mostly individual cusps, as well as a marine reptile unicuspid tooth. Elongated teeth (‘tusks’) occupy the top of the graph, where the region with the highest aspect ratio is occupied by the tusks of the narwhal *Monodon monoceros*, woolly mammoth *Mammuthus primigenius*, African elephant *Loxodonta africana*, and walrus *Odobenus rosmarus*. Most mammal canines have a *Slope* between 0.35 and 0.60 (with a mean standard error *Slope* for each tooth of 0.011). The highest *Slope* is found in the human canine EDJ. Snake fangs have a *Slope* between 0.4 and 0.6 and a high *Aspect Ratio* (above 2.5). All of the *Tyrannosaurus rex* teeth along the row fall within a small range of *Aspect Ratio* and *Slope*. Our examination of tooth shape not only includes extant organisms, but also the greater morphological diversity of extinct organisms, increasing our confidence in the generality of the model [32].

## Discussion

### Power cascade growth is not accounted for by existing models of tooth growth

There are currently two general models that each strive to describe or explain various aspects of tooth development. Enamel knots produce inhibitory signals that prevent new enamel knots forming close to an existing knot [17]. The ‘patterning cascade’ model describes how this inhibition, along with the folding of the epithelial-mesenchyme interface, creates limitations on the size and position of successive cusps during development [33]. First described in seal postcanine teeth, the patterning cascade model has since been extended to primate molars [34, 35]. The second model, the ‘inhibitory cascade’, describes the relative size of sequentially produced teeth, such as molars, as a linear change in size along a tooth row [2, 36]. Neither of these models addresses the shape of cusps.

The power cascade model proposed here is a third general model of tooth development complementary to the two existing models, indicating how the shapes of unicuspid teeth and individual cusps are generated. After determination of cusp shape by the power cascade model, we postulate that cusp spacing is dictated by inhibition of enamel knots according to the patterning cascade [33], and number of cusps is controlled by the number of enamel knots that can fit in the total area of the tooth. The sizes of sequential teeth are then directed by the inhibitory cascade [2, 36]. Therefore, cusp shape, cusp number, and tooth size can be simulated according to this trio of models to generate the main features of an entire tooth row.

The power function has been used to represent or measure a limited set of teeth in previous studies, including the tips of shapes designed for mechanical penetration testing [37], using an average *Slope* of 0.5. Felid canine profiles measured using power functions [38] showed that they generally had a *Slope* of ~ 0.55. Both of these studies are consistent with the current findings in many mammal canines, but they did not generalise this pattern to all teeth or cusps.

Detailed developmental computer simulations of tooth morphogenesis have used a 3D reaction-diffusion-like model that calculates bending stresses to form cusps and teeth [39, 40]. This model produces cusp positions that can have morphological variation similar to biological teeth [39, 41]. Here we tested whether the cusp shapes produced by that model conform to the power cascade model. Varying five parameters of the model that simulates the development of ringed seal postcanine teeth [41] shows that most of the cusp shapes produced do not closely resemble the expected power cascade, with *R*^2^ between 0.59 and 0.97 (Additional file 1: Figure S6). Therefore, the power cascade model describes cusp shape (or cross-sectional profile) substantially better than complex in silico models, although this may be a result of the limited number of cells in the simulations.

Given the power of this new model to define the limits of tooth shape in animals, we expanded our focus to compare it with existing models of growth in other morphological systems. Wren’s [19] model of shells growing as a cone bending to form a logarithmic spiral has since been used to model shells and teeth [6, 7, 20]. Starting with a cone, a logarithmic spiral is generated when one side grows faster than the other, causing the cone to bend to one side (Fig. 1b; Additional file 1: Figure S7d). A mechanism to generate a logarithmic spiral is the unequal growth rates of the two sides A and B. Logarithmic spirals have a formula in polar coordinates *S = a e*^*b θ*^, where *θ* is the angle of rotation around the origin, *S* is the resulting radius of the logarithmic spiral, and *a* and *b* are parameters affecting the size and rate of expansion of the spiral, respectively (Additional file 1: Figure S7a). The radius of the shell opening expands linearly with the angle of rotation (*Radius* = *c θ*, where *c* is a parameter affecting the rate of growth of the shell opening), which creates a cone spiralling around the central axis (Additional file 1: Figure S7b). This model was used to generate shell shapes of many types by modifying relative rates of growth [7, 42, 43].

**Fig. 6:**
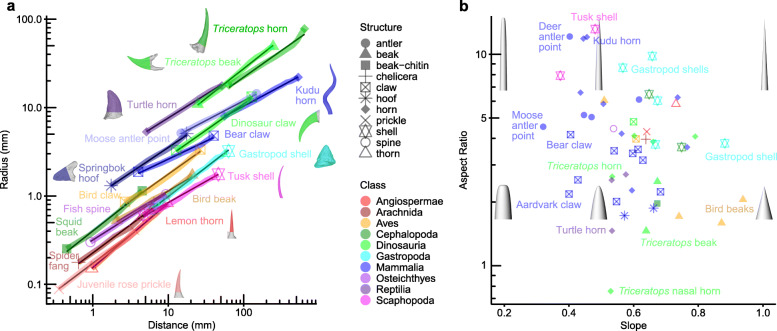
Pointed structures in vertebrates, invertebrates, and plants follow the power cascade model. **a** Log *Distance* vs log *Radius* for structures found in animal and plant classes. **b** Occupation of non-tooth structures in *Slope-Aspect Ratio* morphospace. Note that none of the structures, including shells, fall at *Slope* = 1 where the shape is a cone. Bird beak and gastropod shell labels indicate different specimens in the two graphs

The Raup [7] shell equation describes shell growth using a cone, which is the shape where *Slope* = 1 in our *Log Distance-Radius* plots (Fig. 6; Additional file 1: Figure S2). If this model accurately describes shell growth, all shells should fall on the right-hand edge of the morphospace in Fig. 6. The shells of molluscs (scaphopod *Dentalium* sp. and gastropod *Bembicium auratum*) and cephalopods (nautilus *Nautilus pompilius* and ram’s head squid *Spirula spirula*) each apparently form logarithmic spirals, but follow the power cascade with *Slope* between 0.37 and 0.88 (Fig. 6). This shows that power cones can bend to form logarithmic spirals in an analogous manner to that first proposed by Wren [19] for cones (a specific power cone; Fig. 1d). It also establishes that not all shell shapes can be generated by the existing model of development [7]. In order to accommodate such shapes, the Raup [7] model must have the *Slope* parameter added, such that *Radius* = *c θ*^*Slope*^. In the first description of the shell growth model, Raup [44] assumes that ‘the rate of expansion of the generating curve is approximately constant’, i.e. *Slope* = 1, and so this parameter was not included in his model. In contrast, Thompson [6] suggested that the growth may not be constant in some shells but in fact vary ‘in accordance with some simple law’, and Ackerly [45] showed that for some shells there is an allometric component to the change in radius. Our power cascade model accounts for this important feature of growth.

The long axis of each tooth grows as a logarithmic spiral [6, 46], which can be seen in an extreme form in the curved upper tusks of the babirusa *Babyrousa celebensis*. However, we find that the *Slope* of these tusks (0.25) is considerably less than 1, and therefore, they are not conical (Fig. 5): their high *Aspect Ratio* can make them appear more conical. This means that teeth cannot be modelled by the Raup [7] shell equation. The radius of the circle must change logarithmically with the angle of rotation to form a power cone, rather than a straight-sided cone with *Slope* = 1.

### A general model of growth for horns, claws, spines, beaks, and thorns

Thompson [6] expected that pointed and spiral structures such as horns and claws would follow the same growth pattern as shells, which has been used to model some horn-like structures [47]. If horns grow according to the shell model and are spiralled cones, then their *Slope* parameter will be 1. From measurements of bony horn cores from vertebrates including mammals, non-avian dinosaurs (referred to here as dinosaurs) and reptiles, we have found that *Log Distance-Radius* plots are linear and the *Slope* is typically between 0.4 and 0.8 (Fig. 6; Additional file 1: Figure S8), demonstrating that they do follow the power cascade but are not growing according to the original conical shell model.

Other structures throughout vertebrates also show power cascade growth, including mammal, bird and dinosaur claw and hoof bones (unguals), the bony beaks of birds and dinosaurs, and spines of fish (Fig. 6). Outside vertebrates, the power cascade model is also followed in arthropod fangs and cephalopod beaks. Beyond animals, it is found in thorns and prickles in plants (Fig. 6).

The rose prickle (generally called a thorn) represents an interesting exception. While the concave shape of a mature prickle does not follow the power cascade prediction, a young prickle does (Additional file 1: Figure S9). It appears that the prickle is initially generated following the power cascade growth with *Slope* = 0.6, but then as the stem to which it is attached grows, the base of the prickle is stretched along the long axis of the branch. The result is the typical concave shape of a rose prickle, where only the top half follows the power cascade, not the basal half that has been stretched (Additional file 1: Figure S9). In general, it appears that deviations from the power cascade are more likely in pointed structures controlled by multiple growth processes.

**Fig. 7:**
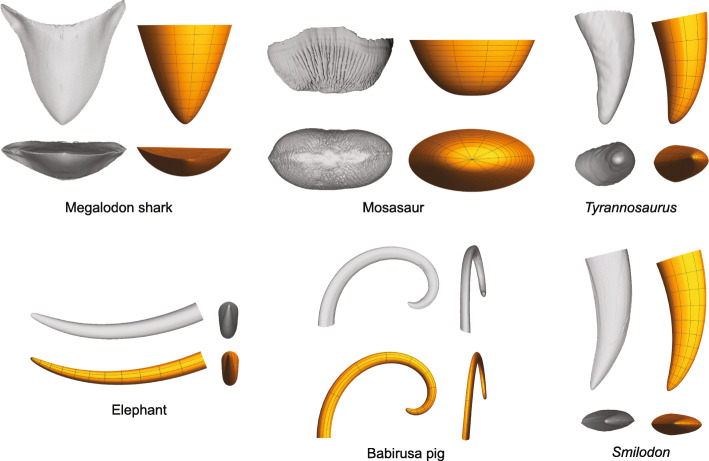
Power spiral (power cascade with a central axis of a logarithmic spiral) can closely emulate real teeth from all vertebrate groups. 3D scan models (grey) and simulated teeth (orange) in two views for megalodon shark *Carcharocles megalodon* (NMV P28786), mosasaur *Globidens alabamensis* (USNM 54078), tyrannosaurid *Tyrannosaurus rex* (UWBM 99000), African elephant *Loxodonta africana* (NMV C30765), babirusa pig *Babyrousa celebensis* (ZMB MAM033677), and sabre-tooth cat *Smilodon fatalis* (LACM HC2000R43)

The power cascade model can be added to the logarithmic spiral model to generate a ‘power spiral’ that can simulate realistic shapes of pointed, curved structures (Additional file 1: Figure S7c). Figure 7 shows some comparisons between real teeth and power spiral models, using both circular cross-sections that would be generated in surfaces of revolution and other cross-sectional shapes (elliptical, lenticular, truncated circle) implemented in a Mathematica notebook (v. 12.0, Wolfram Research Inc., Champaign, IL) available in the Supplementary Information (see also Additional file 1: Figure S10).

The majority of the structures that are closely emulated by the power cascade grow from tip to base, including teeth, horns, thorns, and prickles. These shapes are presumably formed as each addition of material increases the radius by a constant proportion for a proportional increase in length. For example, bovid horns grow from tip to base, increasing in radius down the horn, and they generally follow the power cascade model. In contrast, cervid antlers grow from base to tip, with the growing antler branching, and the antler points being the last structures to form. Despite this directional difference in growth—and the antler starting from a wider base and narrowing towards the tip—antler points also follow the power cascade (Fig. 6). This shows that the proportional growth pattern can act both when increasing the radius of the structure as it cascades downwards from the tip to the base, and also when decreasing the radius to cascade upwards from base to tip. It appears that only the direction of radial growth differs between these two scenarios.

Since many of the structures examined here (including teeth and claws) are used to penetrate food or other materials, it may be argued that selection to maximise penetration ability or structural strength is the cause of the underlying similarity in shape as described by the power cascade model. However, many structures that are not for penetration (such as shells, rounded teeth or backward-curving horns) still follow the power cascade pattern. Given that structures that conform to the power cone can vary from sharp and long to blunt and short, we argue that the most parsimonious explanation for the model fit is an underlying biophysical or developmental mechanism rather than strong selection for shapes that coincidentally fit a power cascade-like pattern. The power cascade generates a base set of allowed variations (Fig. 5), and selection chooses from among these shapes, as occurs with the selection of relative tooth size in hominins according to the inhibitory cascade [36].

### Mechanism and generality of power cascade

The log-log linear pattern of the power cascade can be compared with allometric plots of the relative sizes of body components during growth [20], such as head size versus body size in humans. A linear allometric relationship is produced when two components grow exponentially at different rates. The power cascade relationship shows that there is an allometric relationship within the same structure due to differential growth rates of *Radius* and *Distance*.

**Fig. 8:**
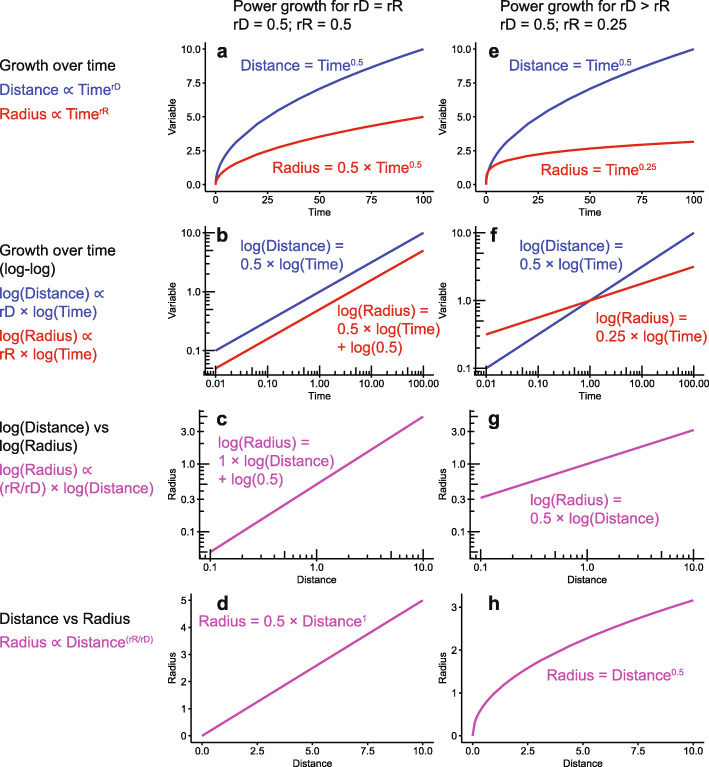
Generation of power cones through allometric growth of *Distance* and *Radius*. From power function growth of both *Distance* and *Radius* (with growth rates *rD* and *rR*, respectively) through time (**a**, **b**, **e**, **f**), the shape of the structure is determined by the ratio of the growth rates (**c**, **g**). Where *rD* = *rR*, a cone is formed (**d**), while where *rD* > *rR*, a curved-sided power cone is generated (**h**). The general equations are shown on the left, while example parameters are shown in the graphs and accompanying equations. See Additional file 1: Supplementary Equations for mathematical derivation

We can demonstrate this growth process by examining power function growth in *Distance* and *Radius* over time (Fig. 8a): *Distance* ∝ *Time*^*rD*^ and *Radius* ∝ *Time*^*rR*^, where *rD* and *rR* are the growth rates for *Distance* and *Radius*, respectively. Power function growth is very common in biology, including for human height [48] and elephant tusks (Fig. 4b). When both axes of the growth over time curves are logged, the plot log(*Distance*) vs log(*Time*) is linear with slope *rD* (similarly for *Radius* and *rR*; Fig. 8b). By solving the log(*Distance*) equation for log(*Time*) and substituting into the log(*Radius*) equation, the relationship between log(*Distance*) and log(*Radius*) through time becomes apparent (Fig. 8c). If *rD* and *rR* are equal, then *Radius* increases linearly with *Distance* (Fig. 8d) and produces a conical shape (with *Log Distance-Radius* power cascade *Slope* of 1). If instead the rates of growth of *Distance* and *Radius* differ (e.g. *rD* = 2*rR*), then the log-log growth over time trajectories will not be parallel (Fig. 8e-f), and the result will be a power cone such as a paraboloid (Fig. 8h). The *Log Distance-Radius* power cascade *Slope* of such a structure will be *rR*/*rD* = 0.5 (Fig. 8g).

Therefore, the power cascade is an expression of allometry as a shape: power cones show unequal power growth within the same structure, or ‘constant differential growth-ratios’ in the terminology of Huxley [20]. The cone is produced through isometric growth between *Distance* and *Radius*, while a power cone results from allometric growth (*rD* ≠ *rR*). The same shapes can also be generated through exponential (as opposed to power) growth of body parts, although this is not commonly found in organisms. Constant differential growth of the two sides of a structure must generate a logarithmic spiral ([20]; Fig. 1b). In the same manner, differential power growth of *Distance* and *Radius* must generate a power cone (Fig. 1c). Both mechanisms could operate at the same time, forming a power cone on a logarithmic spiral, or a power spiral (Fig. 1d).

The power cascade, and likewise the logarithmic spiral, can be seen as ‘dynamical patterning modules’ [49] that generate patterns and structures in metazoans and plants. Despite over three centuries of research [19], the specific molecules driving logarithmic spiral growth are not known (although recent work has begun to reveal some components in gastropod shells [50]). Likewise, the identity of signalling molecules and genes that influence the differential growth of the power cascade very likely must vary widely across animals and plants. Here we show that common growth patterns in animals and plants generate power cones. These shapes may be considered the default family of shapes for pointed structures, meaning they are more likely to independently evolve multiple times and will be a likely source of homoplasy in evolution.

## Conclusions

Since the time of Wren [19], the logarithmic spiral has been considered a fundamental pattern of biological growth, generated by differential growth rates [20]. The power cascade directs the shape of an immense range of structures and likely is as widespread and elementary as the logarithmic spiral in nature. Due to the huge breadth of structures and taxa in which this pattern is found, it appears that the power cascade is a fundamental pattern of growth in myriad organisms [6, 51].

## Materials and methods

### Specimens and 3D scanning

Specimens scanned for this study were sourced from the following institutions or private collections: Museums Victoria, Melbourne, Australia (NMV); Monash University Zoology Research Collection, Melbourne, Australia (MZRC); Australian Museum, Sydney, Australia (AM); American Museum of Natural History, New York, NY, USA (AMNH); Evans EvoMorph Collection, Monash University, Melbourne, Australia (EEM); Natural History Museum of Los Angeles County, Los Angeles, CA, USA (LACM); Natural History Museum UK, London, UK (NHMUK); John Canning, Victoria, Australia (JC); Monash University Integrated Morphology and Palaeontology Laboratory, Melbourne, Australia (MU-IMP); South Australian Museum, Adelaide, Australia (SAMA); Judith Pollock, Melbourne, Australia (JFP); Tasmanian Museum and Art Gallery, Hobart, Australia (TMAG). 3D surface models of teeth were generated using microCT (Zeiss Xradia 520 Versa XRM, Monash University XMFIG X-ray Microscopy Facility for Imaging Geomaterials; Skyscan 1174, Monash University), medical CT (Siemens, Monash Biomedical Imaging), or 3D surface scanning (Laser Design Surveyor 2025; Artec Space Spider). Scans of specimens from the following museums were obtained from researchers or online databases such as MorphoSource and Aves3D: Alabama Museum of Natural History, Tuscaloosa, AL, USA (ALMNH); Ditsong National Museum of Natural History, Pretoria, South Africa (DNMNH); Kronosaurus Korner, Richmond, Queensland, Australia (KK); Museum für Naturkunde, Berlin, Germany (ZMB); Museum of Comparative Zoology, Harvard, MA, USA (MCZ); Natural History Museum, London, UK (NHMUK); Ohio University Vertebrate Collection, Athens, Ohio, USA (OUVC); Royal Ontario Museum, Ontario, Canada (ROM); Smithsonian Institution, Washington, D.C., USA (USNM); Stony Brook University, NY, USA (SBU); University of Leipzig Anatomical Collection, Leipzig, Germany (ULAC); University of Washington Burke Museum, Seattle, USA (UWBM); Yale Peabody Museum, Newhaven, CT, USA (YPM). A total of 200 teeth/cusps from 120 specimens were examined, and 46 specimens for non-tooth structures (see Additional file 1: Tables S1-S5 for sample sizes). CT data were thresholded and/or segmented in Avizo v. 9.6 (Thermo Scientific, Waltham, MA, USA). Surface files were processed in Geomagic Wrap v. 2015 (3D Systems, Rock Hill, SC, USA). The dataset includes all structures that were measured and considered sufficiently complete (essentially unworn and not broken), and no measured structures were excluded from the study for any other reason. We strove to include as wide a taxonomic range as possible, such as teeth from all orders of vertebrates, and from as many families of mammals as possible. Where permitted by institutions/individuals, 3D models are available at [52] on MorphoSource (https://www.morphosource.org/).

### Power cascade shape analysis

For each 3D surface model of a tooth, we estimated a 3D midline through the centre of the tooth from tip to base. First, the midline was approximated by eye in Rhinoceros 3D v. 5.0 or 6.0 (Robert McNeel & Associates, Seattle, WA, USA). At 10 equally spaced locations along the estimated midline, test cross-sections through the tooth surface were generated perpendicular to the estimated midline. The centroid of each test cross-section was calculated, and the final midline was fit as a 3D spline running through these centroids. To measure the rate of radius increase, we placed 10 equally spaced cross-sections perpendicular to its midline (Fig. 2a) and calculated the average radius of each cross-section (*Radius* = √ [cross-sectional area/π]) using Rhinoceros 3D. Cross-sectional area includes all area interior to the external surface of the tooth, including pulp cavity. The use of tooth cross-sectional area perpendicular to the midline essentially removes the effect of the spiral (logarithmic or otherwise) so that our measurements concentrate on the rate of expansion of the radius of the tooth, not the trajectory of the midline in 3D space. All measurement data are included in Additional file 2: Data S1. Circumferential measurements of elephant and mammoth tusks were obtained from the literature [28, 53] and transformed to radius (*Radius* = [circumference/(2π)]). We then plotted log_10_ *Distance* from tip vs log_10_ *Radius*. An ordinary least squares (OLS) linear model was fit to each log *Distance* vs log *Radius* plot, with the *R*^2^ of the regression indicating goodness of fit to the predicted linear power cascade pattern using R Statistical Computing v. 3.5.0 [54] and RStudio v. 1.1.447. Tooth development simulations were generated using the ToothMaker model [40, 41], and parameters and measurement data are in Additional file 3: Data S2. Non-tooth structures were scanned and measured in an analogous manner to teeth, and the measurement data are in Additional file 4: Data S3. The power cascade model was implemented with a logarithmic spiral growth pattern (forming a power spiral) in Mathematica v. 12.0 (Wolfram Research Inc., Champaign, IL), incorporating various cross-sectional shapes (circle, ellipse, lens, truncated circle)—see Fig. 7, Additional file 1: Supplementary Equations and Figure S10.

## Supplementary Information

**Additional file 1: Figure S1.** Vertebrate teeth show power cascade growth. **Figure S2.** Power cascade shapes are characterized as surfaces of revolution for power functions, with variables *Slope*, *Intercept* and *MaxDistance*. **Figure S3.** Alternative sampling intervals along an elephant *Loxodonta africana* NMV C30765 tusk. **Figure S4.** Two ways in which tooth can deviate from linear power cascade: tip offset and missing tip. **Figure S5.** Power cascade shapes are self-similar curves. **Figure S6.** In silico tooth development models do not produce cusps that closely approximate power cascade found in natural teeth. **Figure S7.** Logarithmic spiral, shell model, power cascade model and power spiral model. **Figure S8.** Pointed structures in vertebrates, invertebrates and plants show power cascade growth. **Figure S9.** Prickle growth in roses causes deviation from power cascade growth. **Figure S10.** Power cascade interface implemented in Mathematica for generating biological shapes using power cascade and logarithmic spiral. **Figure S11.** Graphical abstract – Power cascade combined with the logarithmic spiral can generate many biological shapes. **Table S1.** Number of species, specimens and structures in each class for all structures (teeth and non-teeth) measured in this study. **Table S2.** Number of species, specimens and teeth/cusps in each class for all teeth measured in this study. **Table S3.** Number of species, specimens and teeth/cusps in each mammalian order for all teeth measured in this study. **Table S4.** Number of species, specimens and non-tooth structures in each class for all non-tooth structures measured in this study. **Table S5.** Number of species, specimens and structures for each type of structure measured in this study. **Supplementary Discussion.** Resampling of power cascade variables. Effect of tip offset on power cascade linear pattern. **Supplementary Equations.** Derivation of power cascade growth mechanism. Mathematica implementation of power cascade model.

**Additional file 2: Data S1.** Distance and cross-sectional area data for vertebrate teeth, cusps and tooth rows. Taxonomy, specimen number, institution, tooth type/position and measurements of cross-sectional area for 10 distances from tip of tooth.

**Additional file 3: Data S2.** Distance and cross-sectional area data for developmental simulations of seal teeth from model by Savriama et al. [41]. Model parameters and measurements of cross-sectional area for 10 distances from tip of tooth. Starting from the ringed seal model of Savriama et al. [41], parameters were increased and decreased to show the effect of each parameter on tooth shape. The central cusp on each model was measured for the power cascade. Parameters: Act, activator; Boy, buoyancy; Deg, degradation of activator; Egr, epithelial growth; Inh, inhibitor.

**Additional file 4: Data S3.** Distance and cross-sectional area data for non-tooth structures: antlers, beaks, chelicera, claws, hooves, horns, prickles, shells, spine and thorn. Taxonomy, specimen number, institution, structure type and measurements of cross-sectional area for 10 distances from tip of structure.

## Data Availability

All data generated or analysed during this study are included in this published article and its supplementary information files. The 3D scan data that support the findings of this study are available on MorphoSource project Power Cascade (C1206) (https://www.morphosource.org/projects/0000C1206) [52] where permitted by institutions/individuals.
