# Supplementary material for: A universal power law for modelling the growth and form of teeth, claws, horns, thorns, beaks, and shells
Source: BMC Biol. 2021 Mar 30;19:58. doi: 10.1186/s12915-021-00990-w (PMC8008625; doi:10.1186/s12915-021-00990-w)
Supplement: Supplementary file 1 — Additional file 1: Figure S1. Vertebrate teeth show power cascade growth. Figure S2. Power cascade shapes are characterized as surfaces of revolution for power functions, with variables Slope, Intercept and MaxDistance. Figure S3. Alternative sampling intervals along an elephant Loxodonta africana NMV C30765 tusk. Figure S4. Two ways in which tooth can deviate from linear power cascade: tip offset and missing tip. Figure S5. Power cascade shapes are self-similar curves. Figure S6. In silico tooth development models do not produce cusps that closely approximate power cascade found in natural teeth. Figure S7. Logarithmic spiral, shell model, power cascade model and power spiral model. Figure S8. Pointed structures in vertebrates, invertebrates and plants show power cascade growth. Figure S9. Prickle growth in roses causes deviation from power cascade growth. Figure S10. Power cascade interface implemented in Mathematica for generating biological shapes using power cascade and logarithmic spiral. Figure S11. Graphical abstract – Power cascade combined with the logarithmic spiral can generate many biological shapes. Table S1. Number of species, specimens and structures in each class for all structures (teeth and non-teeth) measured in this study. Table S2. Number of species, specimens and teeth/cusps in each class for all teeth measured in this study. Table S3. Number of species, specimens and teeth/cusps in each mammalian order for all teeth measured in this study. Table S4. Number of species, specimens and non-tooth structures in each class for all non-tooth structures measured in this study. Table S5. Number of species, specimens and structures for each type of structure measured in this study. Supplementary Discussion. Resampling of power cascade variables. Effect of tip offset on power cascade linear pattern. Supplementary Equations. Derivation of power cascade growth mechanism. Mathematica implementation of power cascade model. [file 12915_2021_990_MOESM1_ESM.docx]

**Evans et al. 2021 Additional File 1**

**BMC Biology**

**Supplementary Figures**


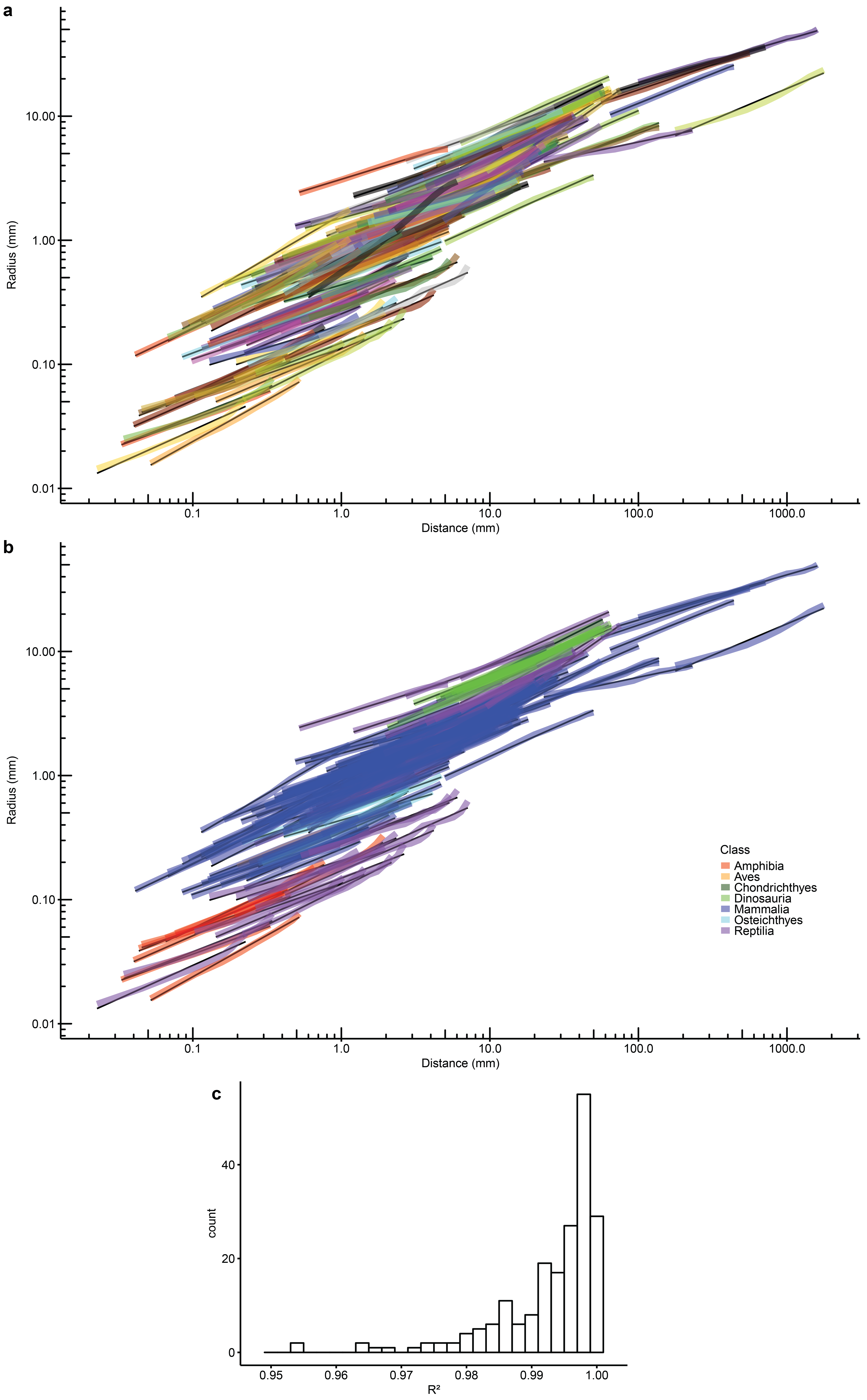


Figure S1 Vertebrate teeth show power cascade growth. a Teeth measured for this study (number of teeth/cusps = 200, number of individuals = 120, number of species = 102, number of orders = 23, number of classes = 7), each tooth with random colour. b Teeth coloured by vertebrate class. Most teeth in bottom left are acrodont reptile teeth (purple), where the base of the tooth is expanded prior to attachment with bone. c Histogram of R^2^ values for power cascade linear model for all teeth plotted in a and b. All R^2^ values below 0.97 are acrodont snake teeth. Mean = 0.9927, median = 0.9961, minimum = 0.9654, maximum = 0.9998.

**
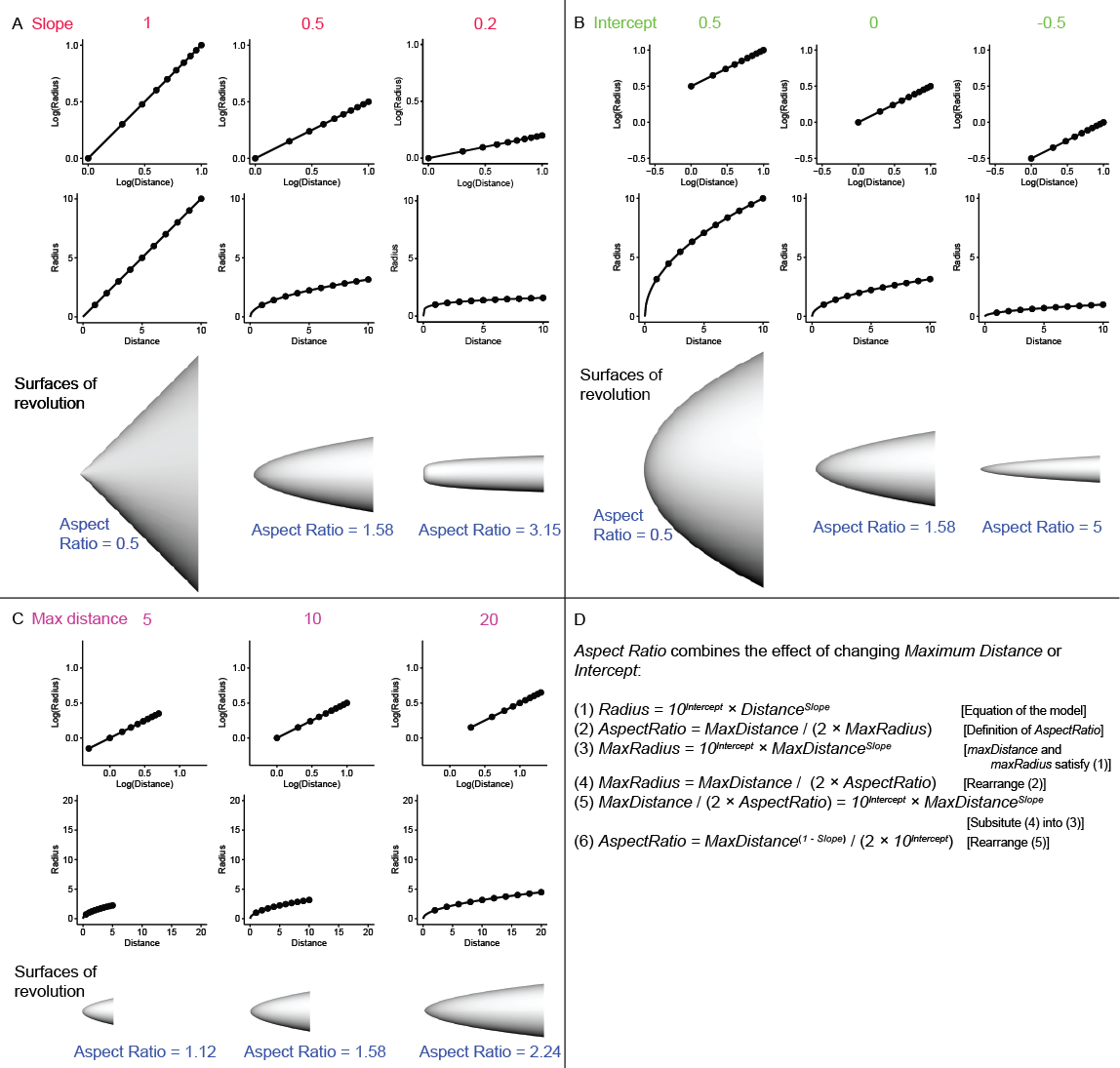
**

Figure S2 Power cascade shapes are characterized as surfaces of revolution for power functions, with variables *Slope*, *Intercept* and *MaxDistance*. a-c Three values for each parameter are shown as log graphs, base 10 graphs and surfaces of revolution. d *Aspect Ratio* combines the effect of changing *Intercept* or *MaxDistance*.


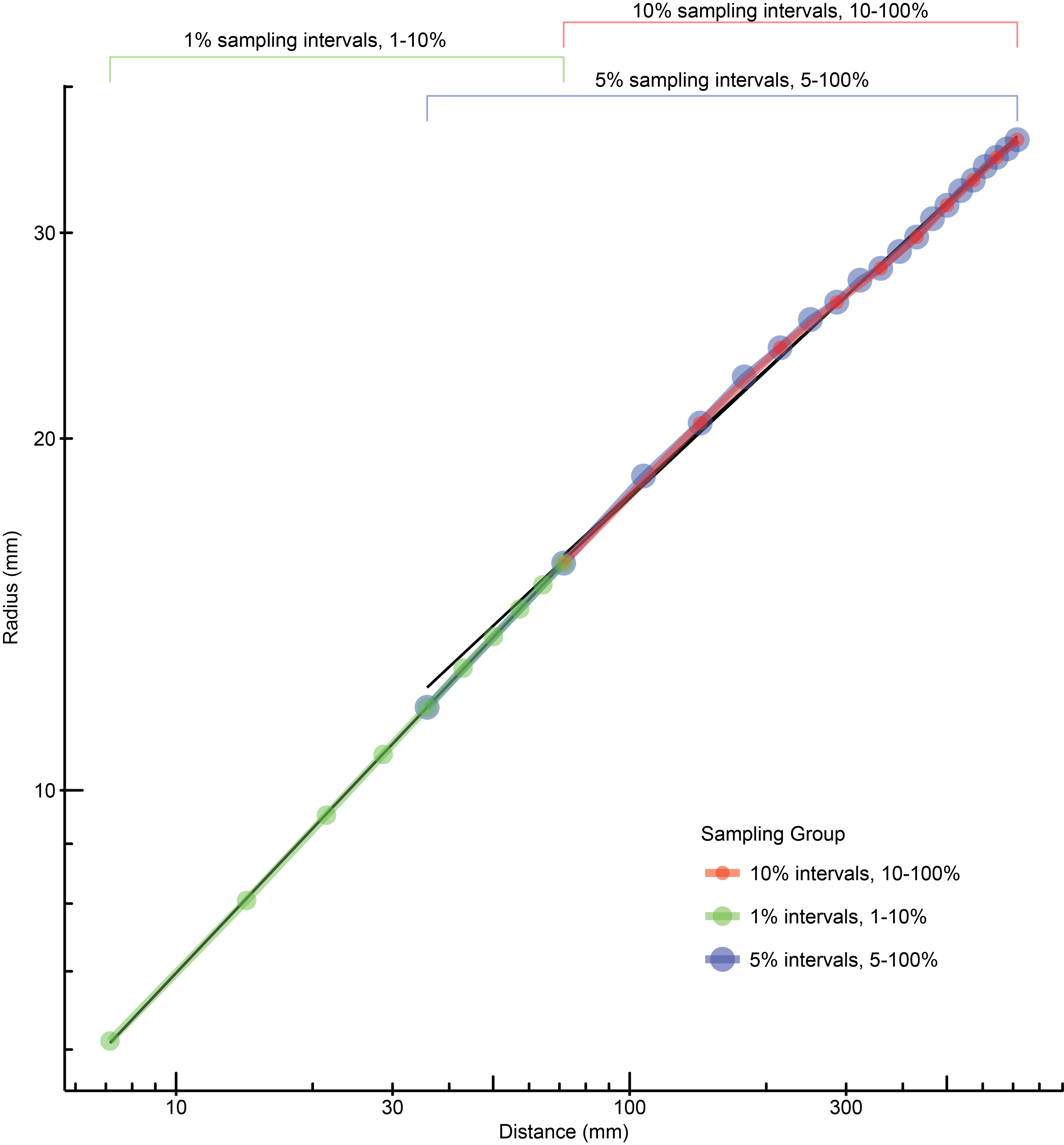


**Figure S3** Alternative sampling intervals along an elephant *Loxodonta africana* NMV C30765 tusk. Sampling the tooth at 10 intervals (10%) or 20 intervals (5%) has very little effect on *Slope* (0.356 and 0.363, respectively) and *Intercept* (0.542 and 0.525, respectively). Also shown is sampling at 1% intervals for the first 10% of the tooth. The x length for the 1-10% is the same as the 10-100% because the minimum length is 1/10 of the maximum length of the sample. Note the slight change in slope at around 25% (179 mm).


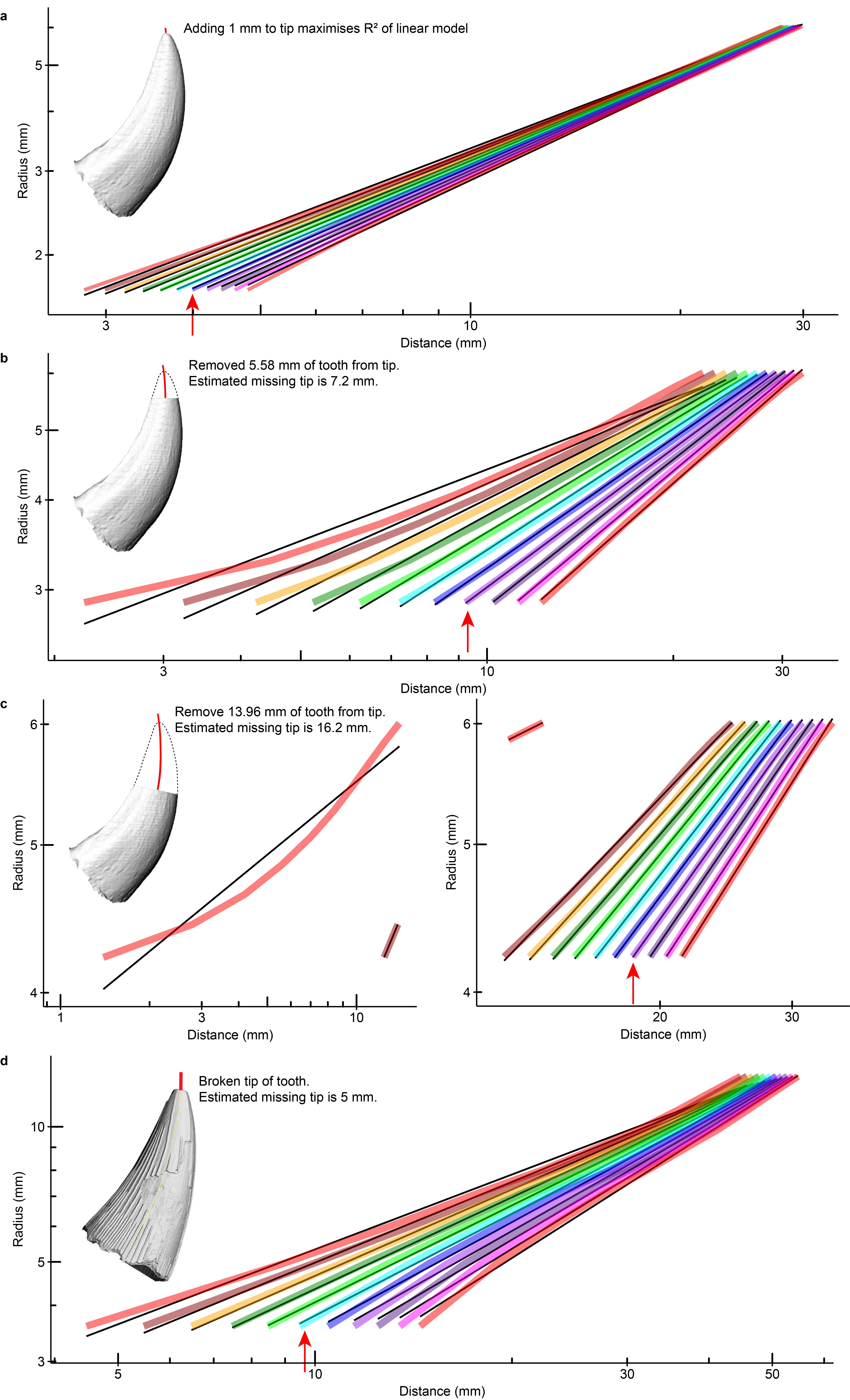


Figure S4 Two ways in which tooth can deviate from linear power cascade: tip offset and missing tip. For each test, the *Distance* vs *Radius* plot was measured, and increments (e.g. of 1 mm) were added to the tip. The incremented *Distance* with the highest R^2^ was the estimated length missing (marked with red arrow). a By adding a tip offset of 1 mm to the length of the leopard seal lower canine (NMV C31561), the R^2^ of the linear model improved from 0.99868 to 0.99996. Increments of 0.2 mm are shown. b 20% of the length of a leopard seal canine was artificially removed from the original length of 27.92 mm. 5.58 mm was missing (original profile shown with dotted lines), and 7.2 mm was estimated missing (shown as red line), giving an overestimate of 5.8%. Taking into account the 1 mm offset, the error would be 2.2%. c 50% of the length was removed (13.96 mm), and 16.2 mm was estimated missing (8.0% overestimate, or 4.4% with 1 mm offset). d Missing tip length was estimated at 5 mm for pliosaur tooth (USNM 16153).


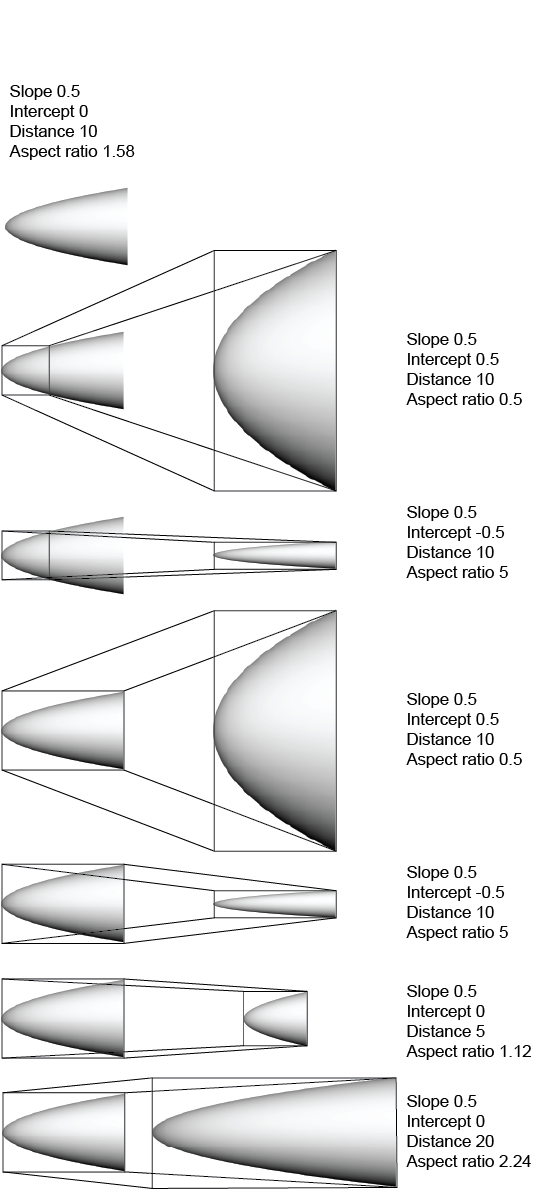


Figure S5 Power cascade shapes are self-similar curves. The self-similar nature of power cascade shapes is illustrated using power cascade shapes of *Slope* = 0.5, such that all shapes with the same *Slope* can be stretched to form any other shape with the same *Slope*. All of the shapes on the left are identical (*Slope* = 0.5, *Intercept* = 0, *Distance* = 10, *Aspect Ratio* = 1.58). Each shape on the right can be made by taking a part or all of the shape on the left and stretching/scaling to new outer dimensions.


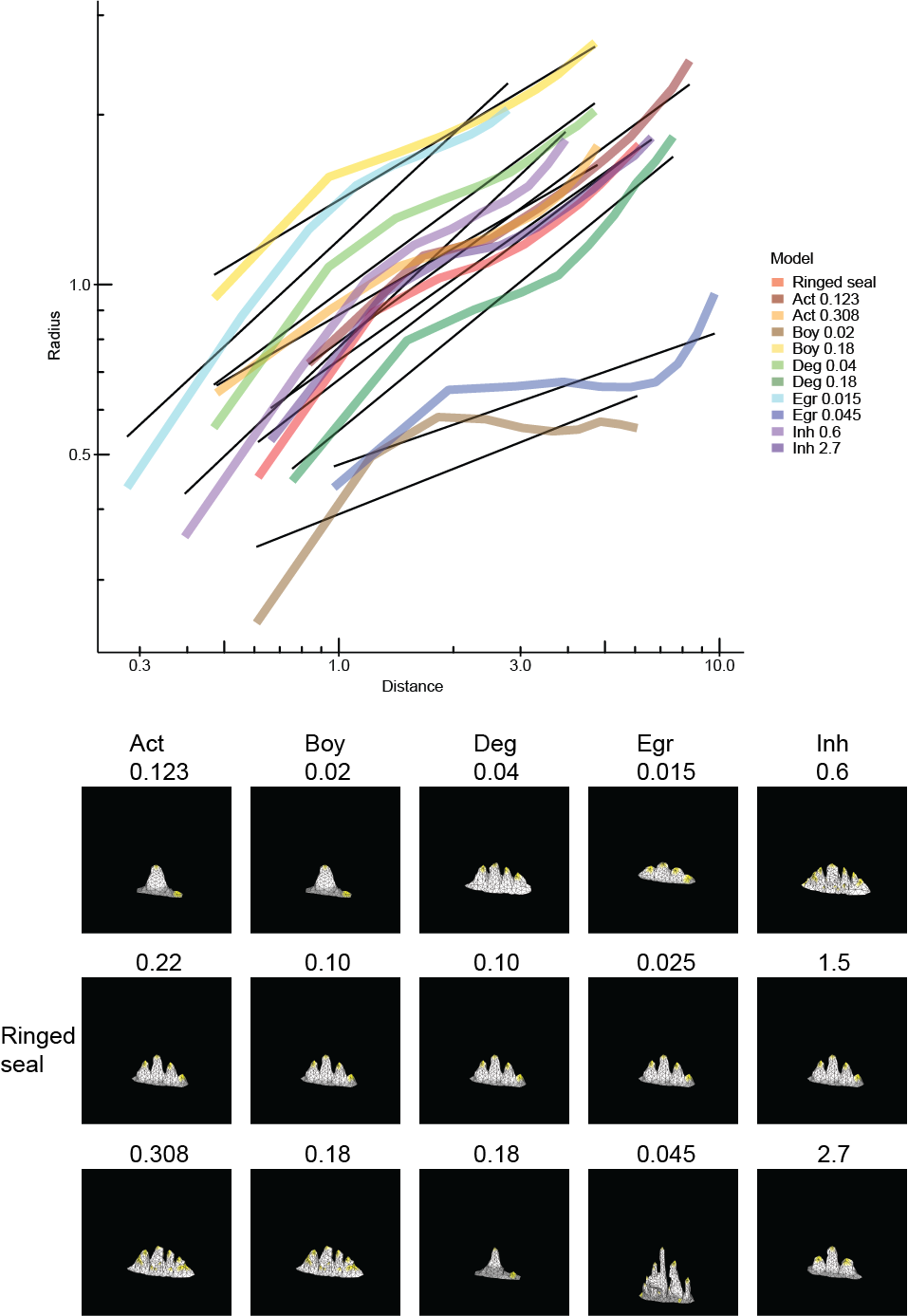


Figure S6 *In silico* tooth development models do not produce cusps that closely approximate power cascade found in natural teeth. Starting from the ringed seal model of Savriama et al. [41] (central row), parameters were increased and decreased to show effect of each parameter on tooth shape. The central cusp on each model was measured for the power cascade. Parameters: Act, activator; Boy, buoyancy; Deg, degradation of activator; Egr, epithelial growth; Inh, inhibitor.


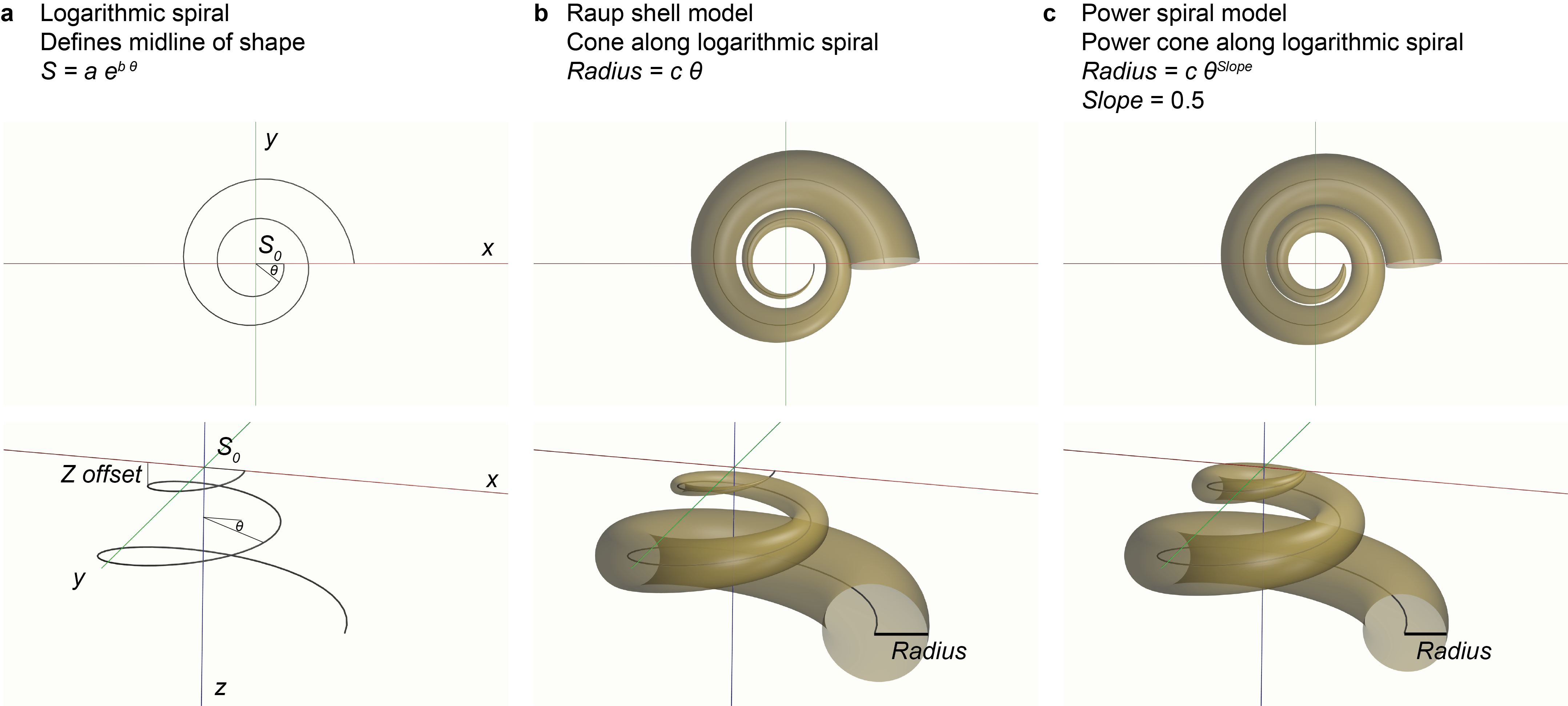


Figure S7 Logarithmic spiral, shell model, power cascade model and power spiral model. a A logarithmic spiral expands outwards as the angle to the origin (*θ*) increases. It can be modified to be a helicospiral by offsetting in the *z* axis. b The Raup shell model generates a cone along the logarithmic spiral, where the *Radius* of the shell opening increases linearly with *θ*. c The power cone generated along a logarithmic spiral creates a power spiral model that more closely simulates teeth, claws and horns.


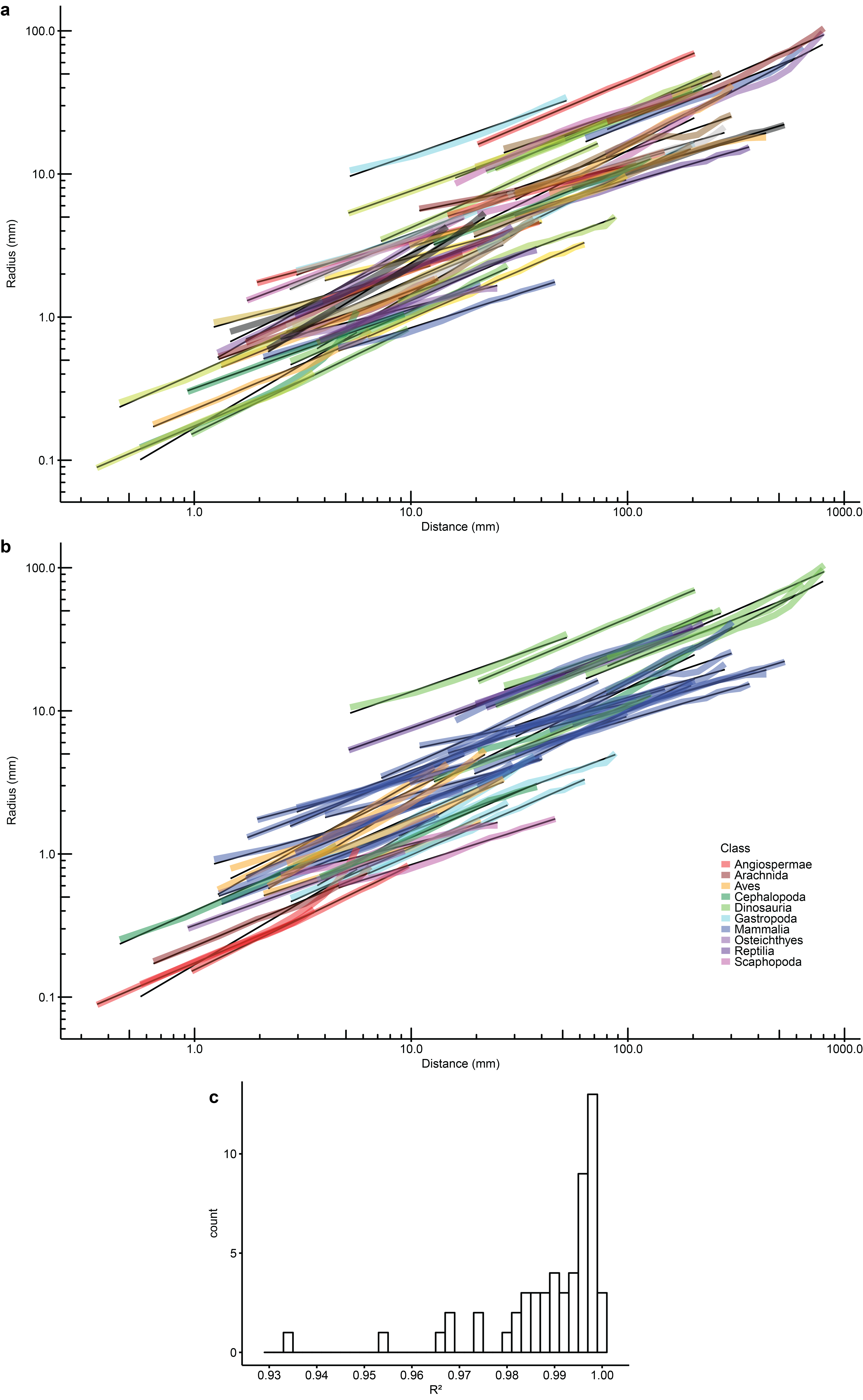


Figure S8 Pointed structures in vertebrates, invertebrates and plants show power cascade growth. a Non-tooth structures measured for this study (number of structures = 51, number of individuals = 45, number of species = 41, number of orders = 24, number of classes = 10), each structure with random colour. b Structures coloured by class. Structures in top right are dinosaur horns (green), where the base of the horn is expanded prior to attachment with bone. c Histogram of R^2^ values for power cascade linear model for all structures plotted in a and b. R^2^ values below 0.96 are mature rose prickle and *Connochaetes taurinus* horn. Mean = 0.9893, median = 0.9937, minimum = 0.9349, maximum = 0.9999.


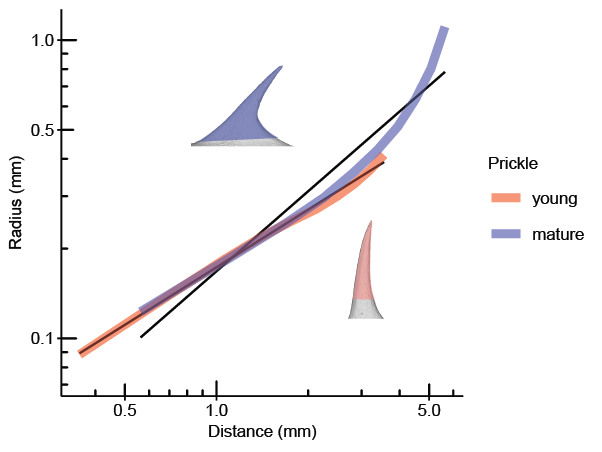


Figure S9 Prickle growth in roses causes deviation from power cascade growth. Initial growth of the prickle (young) shows a linear power cascade pattern (R^2^ = 0.996), while the mature prickle deviates from linear about halfway from the tip (R^2^ = 0.935) after the base of the prickle is stretched along the branch by longitudinal growth of the branch.


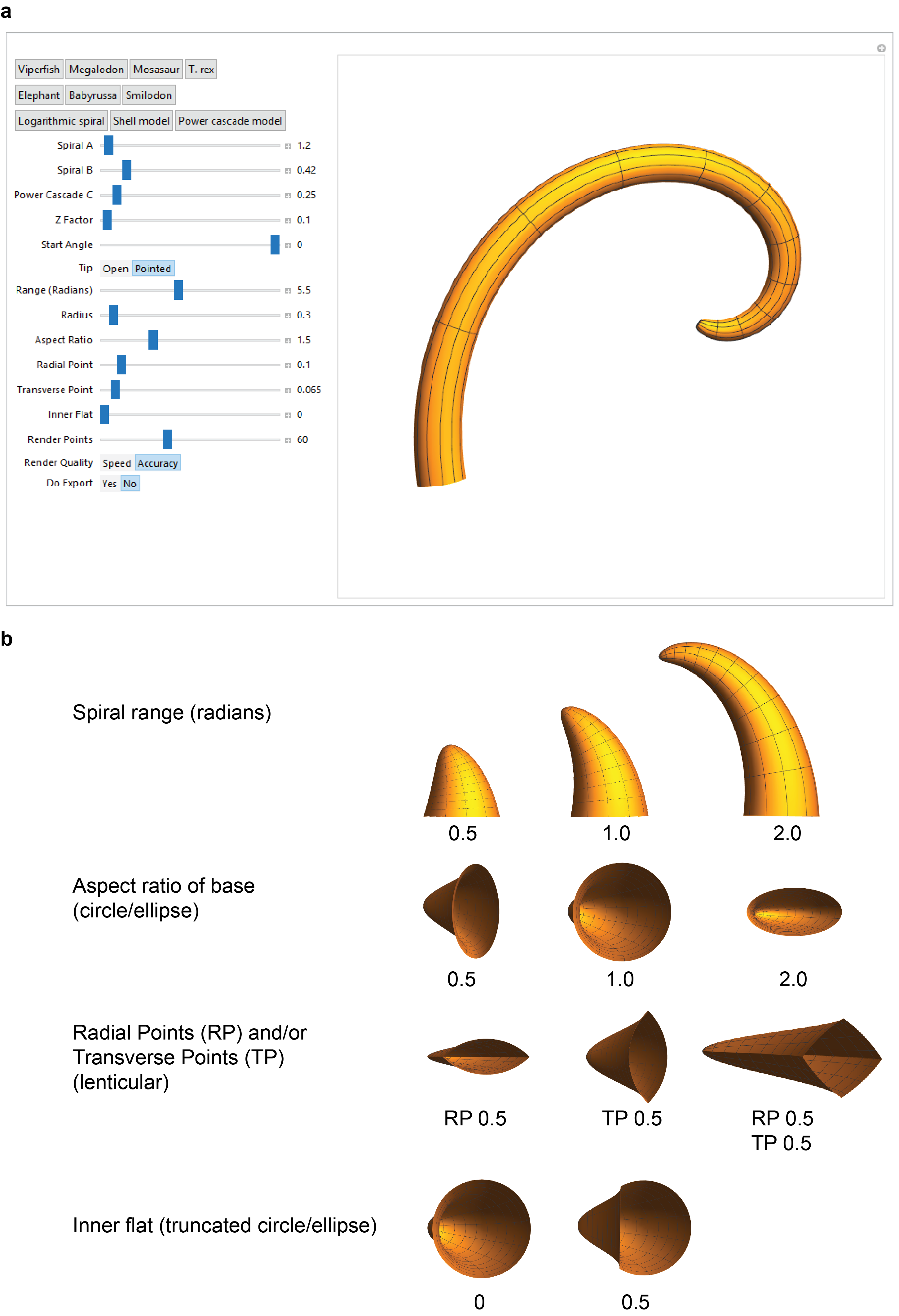


**Figure S10** Power cascade interface implemented in Mathematica (Wolfram Research Inc., Champaign, IL) for generating biological shapes using power cascade and logarithmic spiral. **a** Parameters for the model are shown on the left, along with preset buttons for standard shapes (logarithmic spiral, shell model, power cascade model) and teeth at the top left, while the generated shape (currently showing babirusa tusk) can be viewed and rotated in 3D on the right. **b** Some parameters of the power cascade tooth simulation model, including angular range of the spiral (in radians), aspect ratio of the base circle/ellipse, radial and transverse points generated by circle/ellipse sections, and inner flat region (truncated circle/ellipse).


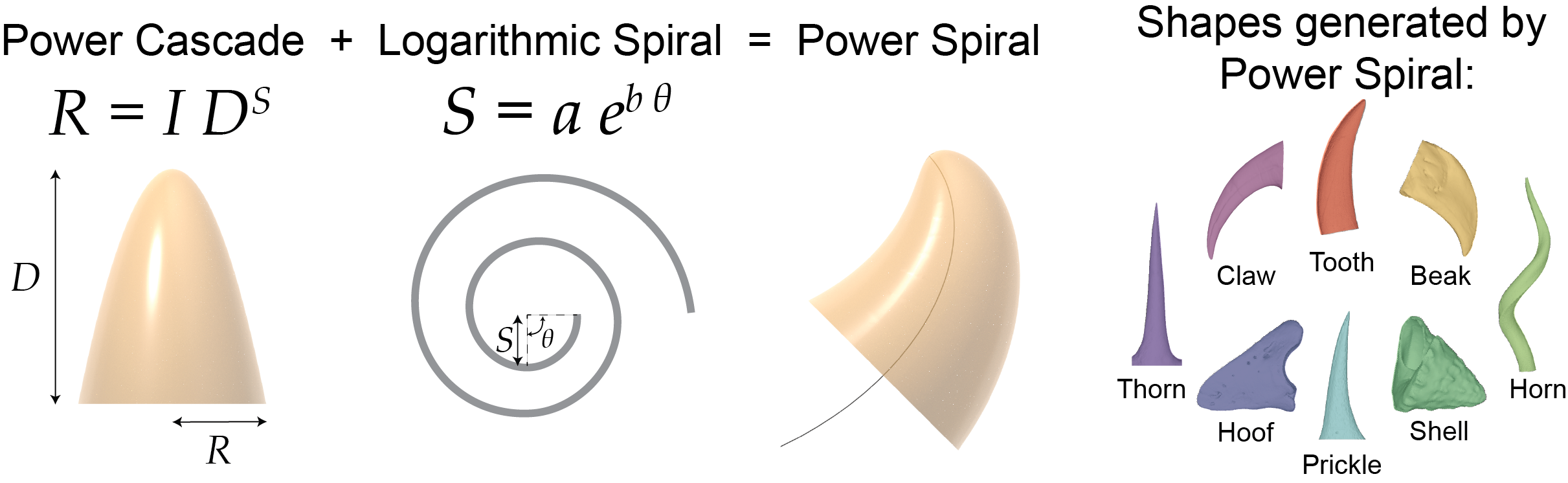


Figure S11 Graphical abstract – Power cascade combined with the logarithmic spiral can generate many biological shapes.

**Supplementary Tables**

**Table S1** Number of species, specimens and structures in each class for all structures (teeth and non-teeth) measured in this study.

| **Class** | **N (Species)** | **N (Specimens)** | **N (Structures)** |
| --- | --- | --- | --- |
| Amphibia | 8 | 8 | 8 |
| Angiospermae | 2 | 2 | 3 |
| Arachnida | 1 | 1 | 1 |
| Aves | 5 | 5 | 6 |
| Cephalopoda | 3 | 3 | 3 |
| Chondrichthyes | 3 | 3 | 3 |
| Dinosauria | 5 | 5 | 20 |
| Gastropoda | 5 | 5 | 5 |
| Mammalia | 79 | 98 | 157 |
| Osteichthyes | 5 | 5 | 7 |
| Reptilia | 25 | 27 | 40 |
| Scaphopoda | 1 | 2 | 2 |
| **Total** | **142** | **164** | **255** |

**Table S2** Number of species, specimens and teeth/cusps in each class for all teeth measured in this study.

| **Class** | **N (Species)** | **N (Specimens)** | **N (Teeth/Cusps)** |
| --- | --- | --- | --- |
| Amphibia | 8 | 8 | 8 |
| Aves | 1 | 1 | 1 |
| Chondrichthyes | 3 | 3 | 3 |
| Dinosauria | 2 | 2 | 12 |
| Mammalia | 60 | 77 | 133 |
| Osteichthyes | 5 | 5 | 6 |
| Reptilia | 23 | 24 | 37 |
| **Total** | **102** | **120** | **200** |

**Table S3** Number of species, specimens and teeth/cusps in each mammalian order for all teeth measured in this study.

| **Order** | **N (Species)** | **N (Specimens)** | **N (Teeth/Cusps)** |
| --- | --- | --- | --- |
| Artiodactyla | 5 | 6 | 6 |
| Carnivora | 26 | 42 | 83 |
| Cetacea | 5 | 5 | 5 |
| Chiroptera | 3 | 3 | 5 |
| Dasyuromorphia | 7 | 7 | 10 |
| Didelphimorphia | 2 | 2 | 2 |
| Diprotodontia | 1 | 1 | 1 |
| Microbiotheria | 1 | 1 | 1 |
| Primates | 8 | 8 | 18 |
| Proboscidea | 2 | 2 | 2 |
| **Total** | **60** | **77** | **133** |

**Table S4** Number of species, specimens and non-tooth structures in each class for all non-tooth structures measured in this study.

| **Class** | **N (Species)** | **N (Specimens)** | **N (Structures)** |
| --- | --- | --- | --- |
| Angiospermae | 2 | 2 | 3 |
| Arachnida | 1 | 1 | 1 |
| Aves | 4 | 4 | 5 |
| Cephalopoda | 3 | 3 | 3 |
| Dinosauria | 3 | 4 | 8 |
| Gastropoda | 5 | 5 | 5 |
| Mammalia | 19 | 21 | 24 |
| Osteichthyes | 1 | 1 | 1 |
| Reptilia | 2 | 3 | 3 |
| Scaphopoda | 1 | 2 | 2 |
| **Total** | **41** | **46** | **55** |

**Table S5** Number of species, specimens and structures for each type of structure measured in this study.

| **Structure** | **N (Species)** | **N (Specimens)** | **N (Structures)** |
| --- | --- | --- | --- |
| Antler | 3 | 3 | 5 |
| Beak | 5 | 6 | 6 |
| Beak-chitin | 1 | 1 | 1 |
| Chelicera | 1 | 1 | 1 |
| Claw | 10 | 10 | 11 |
| Hoof | 2 | 2 | 2 |
| Horn | 12 | 14 | 16 |
| Prickle | 1 | 1 | 2 |
| Shell | 8 | 9 | 9 |
| Spine | 1 | 1 | 1 |
| Thorn | 1 | 1 | 1 |
| Tooth | 102 | 120 | 200 |
| **Total** | **142** | **165** | **255** |

Supplementary Discussion

Resampling of power cascade variables. To investigate the effect of interval sampling along a tooth, an elephant tusk was divided into 20 measurements at 5% intervals along the tooth. 10 of the 20 measurements were randomly selected and linear regression was performed, and the procedure was repeated 100 times. The mean ± s.e.m. (5^th^ and 95^th^ quartiles) of the regression variables were: Intercept: 0.535 ± 0.0026 (0.503, 0.584); Slope: 0.359 ± 0.001 (0.340, 0.371); R^2^: 0.998 ± 0.0001 (0.996, 0.999).

**Effect of tip offset on power cascade linear pattern.** The unworn leopard seal canine gives a *Log Distance-Radius* plot that is slightly concave (Figure S4a). Adding an additional 1 mm to the tip improves the R^2^ of the linear fit to 0.99996. This additional distance may be interpreted as an ‘offset’ of the growth process, such that the tip of the tooth follows a slightly different *Slope* value compared to the rest of the shaft, and this is also found in the elephant tusk (Figure S3). The presence of this developmental ‘offset’ partially explains the relatively low accuracy of the prediction (which would otherwise be less than 5% error; Figure S4), and would limit the ability to accurately predict the original tooth length after wear or fracture.

Supplementary Equations

Derivation of power cascade growth mechanism. Power growth of *Distance* and *Radius* with *Time* result in power cascade shapes being produced. See Fig. 8 for graphs of the equations.

*Distance* = *a*·*Time^rD^* (Equation S1)

*Radius* = *b·Time^rR^* (Equation S2)

Log Equation S1:

log(*Distance*) = log(*a·Time^rD^*) (Equation S3)

log(*Distance*) = log(*a*) *+ rD*·log(*Time*) (Equation S4)

Log Equation S2:

log(*Radius*) = log(*b·Time^rR^*) (Equation S5)

log(*Radius*) = log(*b*) *+ rR*·log(*Time*) (Equation S6)

Solve Equation S4 for log(*Time*):

log(*Time*) = (log(*Distance*) - log(*a*))/*rD* (Equation S7)

Substitute Equation S7 into Equation S6:

log(*Radius*) = log(*b*) *+ rR*·(log(*Distance*) - log(*a*))/*rD* (Equation S8)

log(*Radius*) = log(*b*) *+* (*rR/rD*)·(log(*Distance*) - log(*a*)) (Equation S9)

Column 1 in Fig. 8: *rD* = 0.5, *rR* = 0.5, a = 1, b = 0.5

*Distance* = *Time^0.5^* (Equation S10)

*Radius* = 0.5*·Time^0.5^* (Equation S11)

log(*Distance*) = 0.5·log(*Time*) (Equation S12)

log(*Radius*) = 0.5·log(*Time*) + log(0.5) (Equation S13)

log(*Radius*) = log(0.5) *+* 0.5/0.5 × (log(*Distance*) - log(1)) (Equation S14)

log(*Radius*) = log(*Distance*) + log(0.5) (Equation S15)

*Radius* = 0.5 × *Distance*^1^ (Equation S16)

Column 2 in Fig. 8: *rD* = 0.5, *rR* = 0.25, a = 1, b = 1

*Distance* = *Time^0.5^* (Equation S17)

*Radius* = *Time^0.25^* (Equation S18)

log(*Distance*) = 0.5·log(*Time*) (Equation S19)

log(*Radius*) = 0.25·log(*Time*) (Equation S20)

log(*Radius*) = log(1) *+* 0.25/0.5 × (log(*Distance*) - log(1)) (Equation S21)

log(*Radius*) = 0.5 × log(*Distance*) (Equation S22)

*Radius* = *Distance*^0.5^ (Equation S23)

**Mathematica implementation of power cascade model.** Mathematica notebook (v. 12.0, Wolfram Research Inc., Champaign, IL) code for generating power cascade models simulating teeth and other structures. See Fig S7 for image of graphical user interface.

logAngle[b_]:=logAngle[b]=ArcTan[1/b];

tanSpiral[a_,b_,zOff_][t_]:=tanSpiral[a,b,zOff][t]= Append[AngleVector[logAngle[b] +t ], zOff];

normSpiral[a_,b_,zOff_][t_]:=normSpiral[a,b,zOff][t]=-RotationTransform[ArcTan[zOff],-tanSpiral[a,b,zOff][t]][Append[AngleVector[logAngle[b]+t-π/2 ],0]];

binormSpiral[a_,b_,zOff_][t_]:=binormSpiral[a,b,zOff][t]=Normalize[Cross[tanSpiral[a,b,zOff][t],normSpiral[a,b,zOff][t]]];

mapSpiralNew[γ_,r_, a_, b_, zOff_,cx_, cy_][t_?NumberQ, θ_]:=γ[t]+r[t]cx[θ]normSpiral[a,b,zOff][t]+r[t]cy[θ]binormSpiral[a, b,zOff][t];

lengthFactor[a_, b_] := lengthFactor[a, b] = a*Sqrt[1 + b^2]/b;

logSpiralLength[a_, b_, s_] := logSpiralLength[a, b, s] = lengthFactor[a, b] *Exp[b*s];

curveLengthExact[a_,b_,start_,s_]:=curveLengthExact[a,b,s]=logSpiralLength[a, b, s]- logSpiralLength[a, b, start];

logSpiral[a_,b_,zOff_,sizeGrowth_][t_]:={a Exp[b t]Cos[t], a Exp[b t]Sin[t],zOff*sizeGrowth[t]};

pointCircle[t_,x_,y_]:=pointCircle[t,x,y]=

If[And[x == 1, y== 1],t,

If[t<= π,If[t<= π/2,

t*x*y+(π/2)* (1-x*y)*(1-x)/(2-x-y),

(t - π/2)*x*y+(π/2)* (1-x*y)*(1-y)/(2-x-y)+ π/2],

If[t<= 3*π/2,

(t - π)*x*y +(π/2)* (1-x*y)*(1-x)/(2-x-y)+ π,

(t - 3*π/2)*x*y +(π/2)* (1-x*y)*(1-y)/(2-x-y)+3* π/2]]];

Manipulate[

Module[{zDistFactor, dist, flatDist, sizeGrowth, crossFactor,

crossX, crossY, cross, crossShape, tsl, model},

zDistFactor := Sqrt[1 + zOff^2];

If[openTip == 1,

flatDist[x_] := flatDist[x] = logSpiralLength[a, b, x],

flatDist[x_] := flatDist[x] = curveLengthExact[a, b, start, x]];

dist[x_] := dist[x] = flatDist[x]*zDistFactor;

(*sizeGrowth[x_]:=1;*)

sizeGrowth[x_] := dist[x]^wc;

crossFactor = 1/ Min[Cos[radPoint*π/2], Cos[tranPoint*π/2]];

crossX[t_] := crossFactor*radius*aspect*Min[1 - flat,

Cos[pointCircle[t, 1 - radPoint, 1 - tranPoint]] +

If[Abs[t - π] <= π/2, 1, -1]*

Cos[pointCircle[π/2, 1 - radPoint, 1 - tranPoint]]

];

crossY[t_] := crossFactor*radius *(

Sin[pointCircle[t, 1 - radPoint, 1 - tranPoint]] +

If[t <= π, -1, 1]*

Sin[pointCircle[0, 1 - radPoint, 1 - tranPoint]]

);

tsl = mapSpiralNew[logSpiral[a, b, zOff, flatDist], sizeGrowth, a,

b, zOff, crossX, crossY];

model =

ParametricPlot3D[

tsl[t, θ] // Evaluate, {t, start, range}, {θ, 0,

2 π}, Boxed -> False, Axes -> False,

AxesOrigin -> {0, 0, 0}, PlotPoints -> Ceiling[renderPoints],

ImageSize -> Large, PlotRange -> Full, Mesh -> 10,

PerformanceGoal -> If[speedMode == 1, "Speed", "Performance"],

ViewPoint -> {0, 0, ∞}]; If[doExport == 1,

SetDirectory[NotebookDirectory[]];

Export[

StringJoin["toothModel",

DateString[{"ISODate", "Hour", "Minute"}], ".ply"], model]];

model],

Row[{Button["Viperfish", a = 3; b = 3; wc = 0.43; zOff = 0;

start = 0; openTip = 2; range = 0.82; aspect = 0.96;

radius = 0.49; radPoint = 0.2; tranPoint = 0; flat = 0],

Button["Megalodon", a = 3; b = 1.68; wc = 0.6; zOff = 0;

start = 0; openTip = 2; range = 0.1; aspect = 1; radius = 0.4;

radPoint = 0; tranPoint = 0.3; flat = 1],

Button["Mosasaur", a = 1; b = 1.18; wc = 0.35; zOff = 0;

start = 0; openTip = 2; range = 0.08; aspect = 0.5; radius = 0.3;

radPoint = 0; tranPoint = 0; flat = 0],

Button["T. rex", a = 1; b = 2.8; wc = 0.51; zOff = 0; start = 0;

openTip = 2; range = 0.9; aspect = 1.3; radius = 0.8;

radPoint = 0.1; tranPoint = 0.1; flat = 0]}],

Row[{Button["Elephant", a = 1; b = 1; wc = 0.36; zOff = 0;

start = 0; openTip = 2; range = 0.9; aspect = 1.14;

radius = 0.07; radPoint = 0; tranPoint = 0; flat = 0],

Button["Babyrussa", a = 1.2; b = 0.42; wc = 0.25; zOff = 0.1;

start = 0; openTip = 2; range = 5.5; aspect = 1.5; radius = 0.3;

radPoint = 0.1; tranPoint = 0.065; flat = 0],

Button["Smilodon", a = 1; b = 1; wc = 0.52; zOff = 0; start = 0;

openTip = 2; range = 0.9; aspect = 2; radius = 0.14;

radPoint = 0.1; tranPoint = 0.1; flat = 0]}],

Row[{Button["Logarithmic spiral", a = 1; b = 0.1; wc = 1;

zOff = .15; start = 0; openTip = 2; range = 4 π; aspect = 1;

radius = 0.0001; radPoint = 0; tranPoint = 0; flat = 0],

Button["Shell model", a = 1; b = 0.1; wc = 1; zOff = .15;

start = 0; openTip = 2; range = 4 π; aspect = 1;

radius = 0.05; radPoint = 0; tranPoint = 0; flat = 0],

Button["Power cascade model", a = 1; b = 0.1; wc = .5; zOff = .15;

start = 0; openTip = 2; range = 4 π; aspect = 1;

radius = 0.2; radPoint = 0; tranPoint = 0; flat = 0]}],

{{a, 1, "Spiral A"}, 0.1, 40, Appearance -> "Labeled"},

{{b, 0.5, "Spiral B"}, 0.02, 3, Appearance -> "Labeled"},

{{wc, 0.5, "Power Cascade C"}, 0.1, 2, Appearance -> "Labeled"},

{{zOff, 0, "Z Factor"}, 0, 5, Appearance -> "Labeled"},

{{start, 0, "Start Angle"}, -50, 0, Appearance -> "Labeled"},

{{openTip, 2, "Tip"}, {1 -> "Open", 2 -> "Pointed"}},

{{range, 2, "Range (Radians)"}, 0.001, 4 π,

Appearance -> "Labeled"},

{{radius, 1, "Radius"}, 0.01, 5, Appearance -> "Labeled"},

{{aspect, 1, "Aspect Ratio"}, 0.1, 5, Appearance -> "Labeled"},

{{radPoint, 0, "Radial Point"}, 0, 0.99, Appearance -> "Labeled"},

{{tranPoint, 0.3, "Transverse Point"}, 0, 0.99,

Appearance -> "Labeled"},

{{flat, 0, "Inner Flat"}, 0, 1, Appearance -> "Labeled"},

{{renderPoints, 60, "Render Points"}, 1, 160,

Appearance -> "Labeled"},

{{speedMode, 1, "Render Quality"}, {1 -> "Speed", 2 -> "Accuracy"}},

{{doExport, 2, "Do Export"}, {1 -> "Yes", 2 -> "No"}},

ControlPlacement -> Left, ContinuousAction -> False]
